# Typical development of synaptic and neuronal properties can proceed without microglia in the cortex and thalamus

**DOI:** 10.1038/s41593-024-01833-x

**Published:** 2025-01-06

**Authors:** Mary O’Keeffe, Sam A. Booker, Darren Walsh, Mosi Li, Chloe Henley, Laura Simões de Oliveira, Mingshan Liu, Xingran Wang, Maria Banqueri, Katherine Ridley, Kosala N. Dissanayake, Cristina Martinez-Gonzalez, Kirsty J. Craigie, Deepali Vasoya, Tom Leah, Xin He, David A. Hume, Ian Duguid, Matthew F. Nolan, Jing Qiu, David J. A. Wyllie, Owen R. Dando, Alfredo Gonzalez-Sulser, Jian Gan, Clare Pridans, Peter C. Kind, Giles E. Hardingham

**Affiliations:** 1 Centre for Discovery Brain Sciences, University of Edinburgh, Edinburgh, UK; 2 Simons Initiative for the Developing Brain, University of Edinburgh, Edinburgh, UK; 3 UK Dementia Research Institute at the University of Edinburgh, Edinburgh Medical School, University of Edinburgh, Edinburgh, UK; 4 Mater Research Institute-University of Queensland, Translational Research Institute, Brisbane, Queensland Australia; 5 Centre for Inflammation Research, The Queen’s Medical Research Institute, Institute for Regeneration and Repair, University of Edinburgh, Edinburgh, UK

**Keywords:** Neurotransmitters, Synaptic development, Neuronal development, Microglia

## Abstract

Brain-resident macrophages, microglia, have been proposed to have an active role in synaptic refinement and maturation, influencing plasticity and circuit-level connectivity. Here we show that several neurodevelopmental processes previously attributed to microglia can proceed without them. Using a genetically modified mouse that lacks microglia (*Csf1r*^∆FIRE/∆FIRE^), we find that intrinsic properties, synapse number and synaptic maturation are largely normal in the hippocampal CA1 region and somatosensory cortex at stages where microglia have been implicated. Seizure susceptibility and hippocampal-prefrontal cortex coherence in awake behaving animals, processes that are disrupted in mice deficient in microglia-enriched genes, are also normal. Similarly, eye-specific segregation of inputs into the lateral geniculate nucleus proceeds normally in the absence of microglia. Single-cell and single-nucleus transcriptomic analyses of neurons and astrocytes did not uncover any substantial perturbation caused by microglial absence. Thus, the brain possesses remarkable adaptability to execute developmental synaptic refinement, maturation and connectivity in the absence of microglia.

## Abstract (web summary)

Microglia are proposed to have a role in brain development through synaptic engulfment and paracrine signaling. O’Keeffe et al. show that certain neurodevelopmental processes attributed to microglia can proceed normally even in the absence of these cells.

## Main

The mammalian brain includes complex networks of synaptically connected neurons, supported by glia and the vasculature. During development, there is a period of rapid synapse formation and elimination followed by refinement of synaptic connectivity and maturation of properties of surviving synapses, which is partly dependent on neuronal activity. Cell-autonomous mechanisms of neurons have been proposed to contribute to these processes, including cytoskeletal rearrangements, presynaptic and postsynaptic protein interactions, and membrane trafficking^1^.

However, microglia have additionally been implicated in neuronal development, particularly in events taking place at the synapse^2^. Most studies examining the role of microglia in brain development have focused on three brain regions: hippocampal area CA1; the somatosensory cortex; and the lateral geniculate nucleus (LGN). Such studies examined the local and long-range connections of these brain areas^3–13^. The contribution of microglia has been inferred from analyses of mice globally deficient in certain receptors that are highly (but not exclusively) expressed in microglia (CX3CR1, CR3, TREM2), using complement component knockouts (KOs) (C1Q, C3), or by administering the *Csf1r* antagonist PLX5622 to kill microglia. While the results of these studies present a consistent role for microglia in developmental processes, further testing of this hypothesis is required as no model of microglial perturbation is without significant caveats. For example, the *CSF1R* antagonist PLX5622 has effects on nonmicroglial cells, including circulating macrophages, T and B lymphocytes, and hematopoietic progenitors^14^. Meanwhile, CX3CR1 and the complement system can control metabolic and vascular properties, and aspects of brain development independently of microglial function^15–22^.

Despite these caveats, a substantial body of work nevertheless indicates abnormal brain development when microglial function is altered^3–13^. In this study, we have taken an alternative approach to addressing the role of microglia in brain development by using *Csf1r*^∆FIRE/∆FIRE^ mice that are homozygous for the deletion of a promoter element (Fms-Intronic Regulatory Element (FIRE)) in the *Csf1r* gene^23^. These mice lack microglia throughout life, while still possessing circulating monocytes and other central nervous system (CNS)-resident (for example, perivascular) macrophages^23^. By focusing on developmental milestones and brain regions presumed disrupted by the presence of abnormal microglia, we provide a complementary approach of studying these processes in the absence of microglia.

## Results

### CA1 dendritic and synaptic properties in the absence of microglia

**Extended Data Fig. 1:**
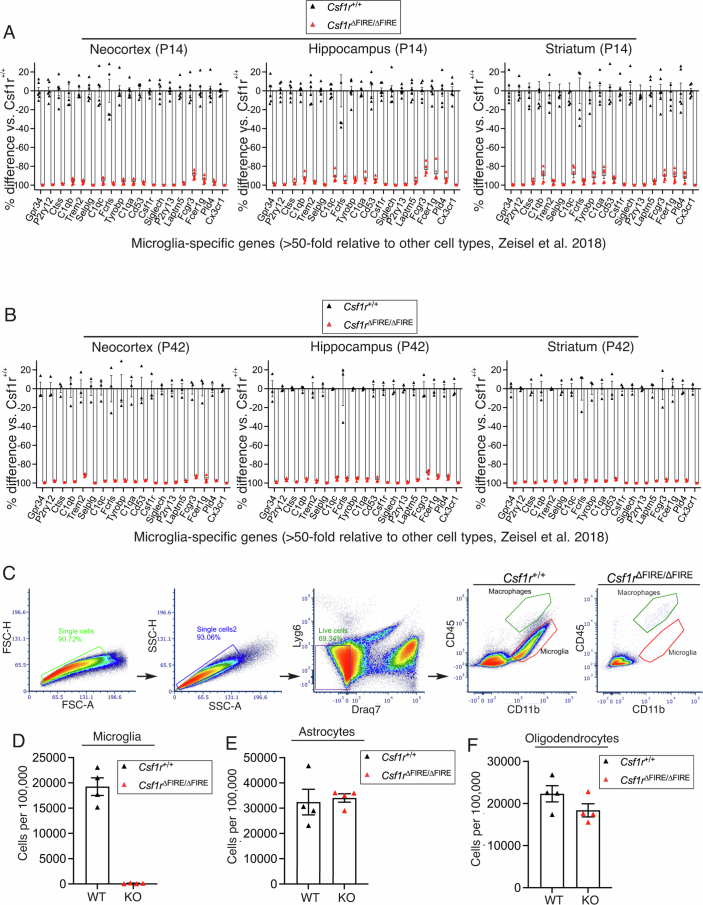
Molecular and anatomical characterisation of Csf1r^ΔFIRE/ΔFIRE^ mice during early development. All data are shown as mean ± s.e.m. and all statistical tests are two-sided **a, b)** RNA sequencing data showing the relative expression of microglia-specific genes in the Csf1r^ΔFIRE/ΔFIRE^ compared to Csf1r^+/+^ for neocortex (left), hippocampus (middle) and striatum (right), at P14 (A) or P42 (B). Genes were selected based on previously published single-cell sequencing data (Ziesel et al., 2018) that described 265 subclusters in the CNS and PNS. To define microglia-enriched genes, we calculated the fold-enrichment of expression in the ‘MGL1’ subcluster relative to the next highest expressing non-immune cell subcluster, requiring an average of ≥1 FPKM in wild-type mice. Data relating to genes with a microglial enrichment of ≥50-fold are shown. Sample size at P14: 6 Csf1r^+/+^ mice, 6 Csf1r^ΔFIRE/ΔFIRE^ mice (3 males and 3 females for both). Sample size at P42: 3 Csf1r^+/+^ mice, 4 Csf1r^ΔFIRE/ΔFIRE^ mice. **c-f)** Cells from the neocortex of P14 mice of the indicated genotypes were analysed by flow cytometry (see Methods). **(c)** shows the sequential gating strategy and example plots of CD11b (x-axis) and CD45 (y-axis) illustrating the loss of CD11b^+^/CD45^low^ microglia in mice, with far less abundant CD11b^+^/CD45^high^ border-associated macrophages retained, (d-f) shows quantitation of microglia, astrocytes and oligodendrocytes (*n* = 4 per genotype).

**Extended Data Fig. 2:**
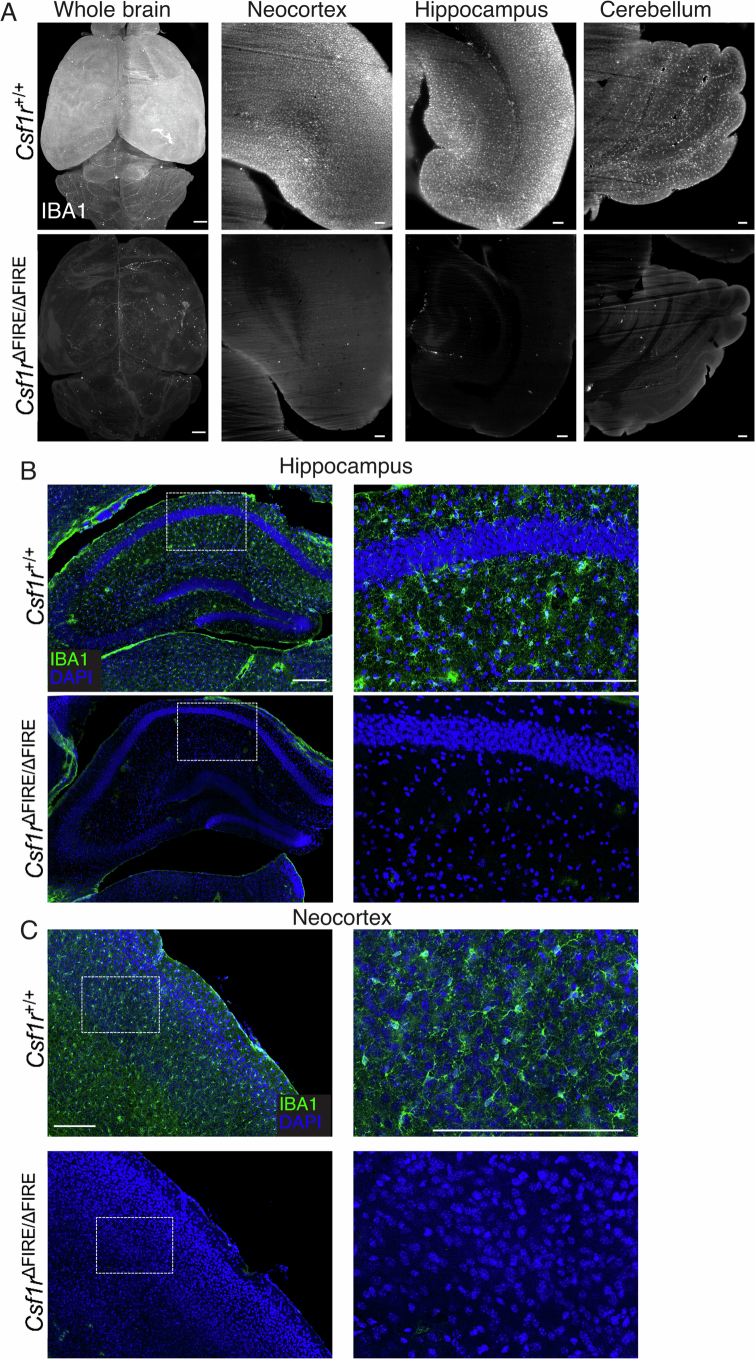
Loss of IBA1-positive microglia in the *Csf1r*^ΔFIRE/ΔFIRE^ mouse. **a**) Example images (representative of 3 repeats) from whole-brain imaging performed using iDISCO. Brains were labelled for IBA1 (greyscale) for *Csf1r*^+/+^(upper) and *Csf1r*^ΔFIRE/ΔFIRE^ (lower) mice at P28. Flattened volume images are shown for the whole brain (left), neocortex (middle left), hippocampus (middle right), and cerebellum (right). Note the prominent labelling for Iba1 in *Csf1r*^+/+^ brains, but not *Csf1r*^ΔFIRE/ΔFIRE^ brains. Scale bars: 500 µm (left, whole brain), 100 µm (middle, right, sub-regions). Note that the tiny amount of residual IBA1 staining in the *Csf1r*^ΔFIRE/ΔFIRE^ brains is due to border-associated macrophages^23^. **b, c)** Conventional immunohistochemical analysis of IBA1 expression (green), counterstained with DAPI, in the indicated brain regions of *Csf1r*^+/+^ and *Csf1r*^ΔFIRE/ΔFIRE^ mice at P14. Scale bar: 200 µm. Images are representative of 6 repeats.

**Extended Data Fig. 3:**
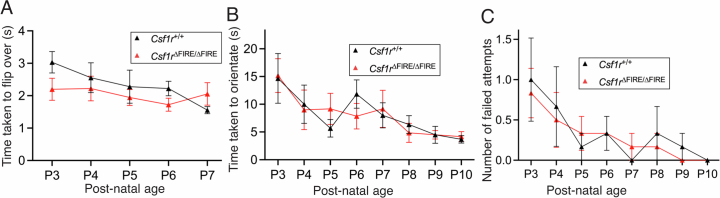
Assessing motor control in young pups. All data are shown as mean ± s.e.m. **a-c)** Mouse pup innate motor reflex testing. (a) shows time to turn over in righting reflex task. (b) and (c) relate to the negative geotaxis task, showing the time to reorientate uphill (b) and number of failed attempts (c); n = 6 mice per genotype (3 males, 3 females).

Before investigating neuronal properties in *Csf1r*^∆FIRE/∆FIRE^ mice, we confirmed an absence of microglia using RNA sequencing (RNA-seq), flow cytometry and immunohistochemistry (IHC) (Extended Data Figs. 1 and 2). RNA-seq analysis of *Csf1r*^∆FIRE/∆FIRE^ brain tissue at 14 (P14) and 42 (P42) postnatal days showed a depletion of microglia-specific genes, including *C1q*, *Trem2* and *Cx3cr1* (Extended Data Fig. 1a,b). Immunohistochemical analysis of IBA1 expression in optically cleared brains confirmed microglial absence (Extended Data Fig. 2a), as did higher resolution imaging of the hippocampus (HPC) and neocortex (Extended Data Fig. 2b,c). Flow cytometry of dissociated P14 neocortex also confirmed the loss of CD11b^+^/CD45^low^ microglia (Extended Data Fig. 1c,d), but retention of (far less numerous) CD11b^+^/CD45^high^ macrophages (Extended Data Fig. 1c) and other glia (Extended Data Fig. 1e,f). *Csf1r*^∆FIRE/∆FIRE^ mice have normal body and brain weight^23^, avoiding confounders of other microglia-deficient models, such as premature death, bone abnormalities and altered CNS macrophages and monocytes^14,24,25^. *Csf1r*^∆FIRE/∆FIRE^ pups appeared healthy and developed typical innate motor skills, as tested by righting reflex and negative geotaxis tasks (Extended Data Fig. 3a–c).

**Fig. 1:**
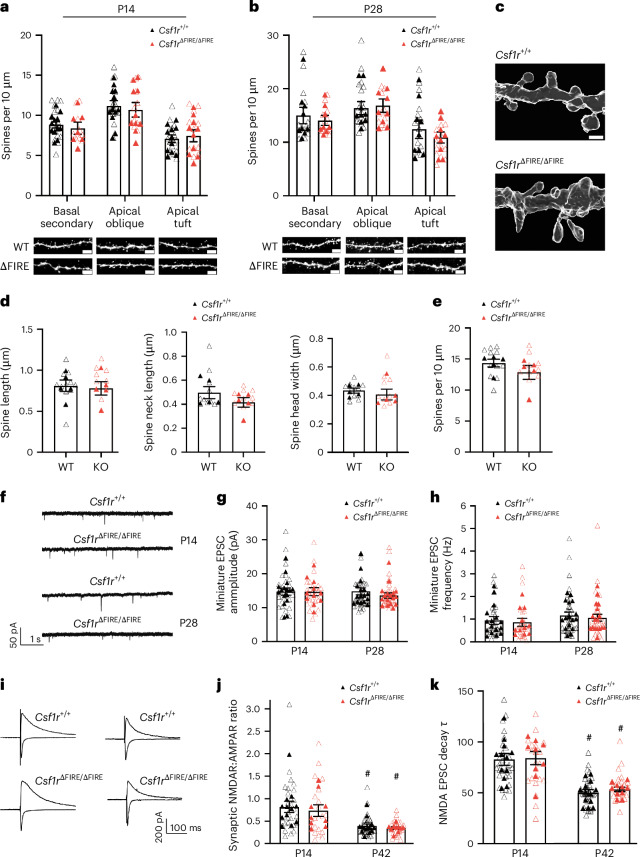
Absence of brain microglia does not alter hippocampal synaptic density or properties. **a**, CA1 spine density (P14). Basal secondary: (*F*_(1,17)_ = 0.825, *t*_(17)_ = 0.908, *P* = 0.377, nested Student’s *t*-test, *n* = 12 *Csf1r*^+/+^ (five male mice, seven female mice) and *n* = 7 *Csf1r*^ΔFIRE/ΔFIRE^ mice (five male mice, two female mice)). Apical oblique: (*F*_(1,20)_ = 0.257, *t*_(20)_ = 0.507, *P* = 0.617, nested Student’s *t*-test, *n* = 12 *Csf1r*^+/+^ (five male mice, seven female mice) and *n* = 10 *Csf1r*^ΔFIRE/ΔFIRE^ mice (six male mice, four female mice)). Apical tuft: (*F*_(1,19)_ = 0.356, *t*_(19)_ = 0.597, *P* = 0.558, nested Student’s *t*-test, *n* = 11 *Csf1r*^+/+^ (five male mice, six female mice) and *n* = 10 *Csf1r*^ΔFIRE/ΔFIRE^ mice (six male mice, four female mice)). **b**, Spine density at P28. Basal secondary: (*F*_(1,15)_ = 0.260, *t*_(15)_ = 0.510, *P* = 0.618, nested Student’s *t*-test, *P* > 0.999 Mann–Whitney *U*-test, *n* = 9 *Csf1r*^+/+^ (three male mice, six female mice) and *n* = 8 *Csf1r*^ΔFIRE/ΔFIRE^ mice (four male mice, four female mice)). Apical oblique: (*F*_(1,16)_ = 0.001, *t*_(16)_ = 0.036, *P* = 0.972, nested Student’s *t*-test, *P* > 0.667 Mann–Whitney *U*-test, *n* = 9 *Csf1r*^+/+^ (three male mice, six female mice) and *n* = 9 *Csf1r*^ΔFIRE/ΔFIRE^ mice (five male mice, four female mice)). Apical tuft dendrites: (*F*_(1,16)_ = 0.872, *t*_(16)_ = 0.934, *P* = 0.364, nested Student’s *t*-test, *P* > 0.667 Mann–Whitney *U*-test, *n* = 9 *Csf1r*^+/+^ (three male mice, six female mice) and *n* = 9 *Csf1r*^ΔFIRE/ΔFIRE^ mice (five male mice, four female mice)). Here and throughout the manuscript, the filled symbols represent independent biological replicates (animals) and the open symbols represent technical replicates (multiple cells studied per animal). **c**, Example STED images of CA1 apical oblique dendrites (P28). **d**, STED microscopy of sections of CA1 apical oblique dendrites (P28) and analysis of spine length (left: *t*_(8)_ = 0.297, *P* = 0.774), spine neck length (middle: *t*_(8)_ = 1.26, *P* = 0.244) and spine head width (right: *t*_(8)_ = 0.646, *P* = 0.537); *n* = 5 *Csf1r*^+/+^ (two male mice, three female mice) and *n* = 5 *Csf1r*^ΔFIRE/ΔFIRE^ mice (two male mice, three female mice). **e**, Spine density of CA1 apical oblique dendrites derived from the STED images: *t*_(8)_ = 1.16, *P* = 0.280 (unpaired *t*-test); *n* = 5 *Csf1r*^+/+^ (two male mice, three female mice) and *n* = 5 *Csf1r*^ΔFIRE/ΔFIRE^ mice (two male mice, three female mice). **f**, Miniature EPSCs recorded at P14 (top) and P28 (bottom). **g**,**h**, Amplitude (**g**) of miniature EPSCs at P14 (*F*_(1,25)_ = 0.345, *t*_(25)_ = 0.588, *P* = 0.562, nested Student’s *t*-test) and P28 (*F*_(1,31)_ = 0.363, *t*_(31)_ = 0.603, *P* = 0.551, nested Student’s *t*-test; *P* = 0.345 Mann–Whitney *U*-test). Miniature EPSC frequency (**h**) at P14 (*F*_(1,25)_ = 0.0404, *t*_(25)_ = 0.2010, *P* = 0.842, nested Student’s *t*-test) and P28 (*F*_(1,31)_ = 0.157, *t*_(31)_ = 0.397, *P* = 0.694 nested Student’s *t*-test, *P* = 0.625 Mann–Whitney *U*-test). P14: *n* = 16 *Csf1r*^+/+^mice (seven males, eight females, one unattributable mouse), *n* = 11 *Csf1r*^ΔFIRE/ΔFIRE^ mice (four male mice, four female mice, three unattributable mice); P28: *n* = 16 *Csf1r*^+/+^mice (seven male mice, nine female mice), *n* = 17 *Csf1r*^ΔFIRE/ΔFIRE^ mice (seven male mice, ten female mice). **i**, Example EPSC traces. AMPAR-mediated and NMDAR-mediated EPSCs were recorded at −70 mV and +40 mV respectively. **j**, Quantification of the NMDAR:AMPAR ratios at P14 (*F*_(1,19)_ = 0.110, *t*_(19)_ = 0.332, *P* = 0.743, nested Student’s *t*-test) and P42 (*F*_(1,21)_ = 0.623, *t*_(21)_ = 0.789, *P* = 0.439, nested Student’s *t*-test, *P* = 0.879 Mann–Whitney *U*-test). P14: *n* = 12 *Csf1r*^+/+^ (six male mice, six female mice) and *n* = 9 *Csf1r*^ΔFIRE/ΔFIRE^ mice (six male mice, three female mice); P42: *n* = 13 *Csf1r*^+/+^ (six male mice, seven female mice) and *n* = 10 *Csf1r*^ΔFIRE/ΔFIRE^ mice (five male mice, five female mice). *P* < 0.0001 (main age effect, two-way analysis of variance (ANOVA) on the averages of each of the animals). # indicates Šídák’s multiple comparisons test: *P* = 0.0022 (*Csf1r*^+/+^) and 0.0158 (*Csf1r*^ΔFIRE/ΔFIRE^). **k**, Quantification of the decay constant of NMDAR-mediated EPSCs at P14 (*F*_(1,19)_ = 0.0008, *t*_(19)_ = 0.027, *P* = 0.978, nested Student’s *t*-test) and P42 (*F*_(1,21)_ = 0.737, *t*_(21)_ = 0.859, *P* = 0.400, nested Student’s *t*-test). P14: *n* = 12 *Csf1r*^+/+^ (six male mice, six female mice) and *n* = 9 *Csf1r*^ΔFIRE/ΔFIRE^ mice (five male mice, six female mice); P42: *n* = 13 *Csf1r*^+/+^ (six male mice, seven female mice) and *n* = 10 *Csf1r*^ΔFIRE/ΔFIRE^ mice (five male mice, five female mice). *P* < 0.0001 (main age effect, two-way ANOVA performed on the averages of each of the animals). # indicates Šídák’s multiple comparisons test: *P* < 0.0001 (*Csf1r*^+/+^) and 0.0002 (*Csf1r*^ΔFIRE/ΔFIRE^). All data are shown as the mean ± s.e.m.; all statistical tests are two-sided. **a**, Scale bar, 3 µm. **c**, Scale bar, 700 nm. Source data

**Extended Data Fig. 4:**
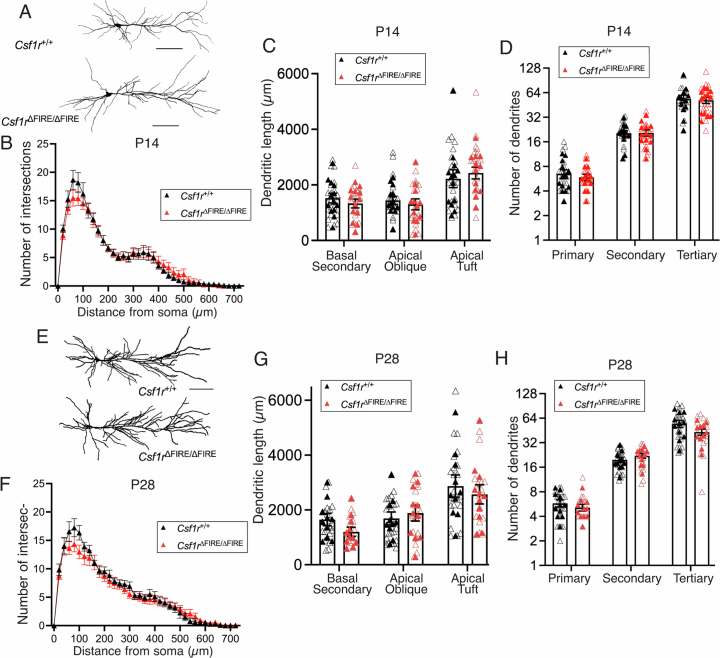
Absence of microglia during neurodevelopment does not lead to changes in CA1 pyramidal cell gross morphology. All data are shown as mean ± s.e.m. and all statistical tests are two-sided. **a)** Example 2D reconstructions of the somato-dendritic axis of dye-filled CA1 pyramidal cells from *Csf1r*^+/+^(upper) and *Csf1r*^ΔFIRE/ΔFIRE^ (lower) P14 mice. Scale bars: 100 µm. **b**) Sholl analysis of reconstructed P14 CA1 pyramidal cell dendrites from *Csf1r*^+/+^ (n = 13 mice) and *Csf1r*^ΔFIRE/ΔFIRE^ (n = 13 mice). We observed no difference of Sholl distribution between *Csf1r*^+/+^ (black) and *Csf1r*^ΔFIRE/ΔFIRE^ (red) mice at P14 (F_(1,24)_ = 0.038, *P* = 0.847, 2-way ANOVA [genotype]); F_(36,864)_ = 0.826, *P* = 0.758 [genotype_x_dendrite interaction]); n = 13 *Csf1r*^+/+^ (n = 13 mice, 5 m/8 f) and 13 *Csf1r*^ΔFIRE/ΔFIRE^ mice (6 m/7 f). **c**) No difference in the length of difference dendritic types for reconstructed CA1 pyramidal cells at basal secondary, apical oblique, or apical tuft dendrites. Basal Secondary: F_(1,24)_ = 0.596, *t*_(24)_ = 0.772, *P* = 0.448, nested Student’s t-test; Apical oblique: F_(1,24)_ = 1.161, t_(24)_ = 1.077, *P* = 0.292, nested Student’s t-test; Apical tuft: F_(1,24)_ = 1.55, t_(24)_ = 1.245, *P* = 0.225, nested Student’s t-test. **d**) No difference in number of dendrites for CA1 pyramidal cells (P14) was observed at primary, secondary or tertiary dendrites. Primary: F_(1,24)_ = 0.621, t_(24)_ = 0.788, *P* = 0.439, nested Student’s t-test; Secondary: F_(1,24)_ = 0.143, t_(24)_ = 0.378, *P* = 0.709, nested Student’s t-test; Tertiary: F_(1,24)_ = 0.020, t_(24)_ = 0.141, *P* = 0.889, nested Student’s t-test. **e**) Reconstructions of CA1 pyramidal cells from P28 mice. **f**) No difference in Sholl distributions of CA1 pyramidal cell dendrites at P28 from *Csf1r*^+/+^ and *Csf1r*^ΔFIRE/ΔFIRE^ mice; F_(1,22)_ = 0.929, *P* = 0.346, 2-way ANOVA [genotype]; F_(36,792)_ = 0.905, *P* = 0.631 [genotype X distance interaction]. **g**) There was no change in the lengths of different dendrite types of CA1 pyramidal cells at P28 at basal secondary, apical oblique, or apical tuft dendrites. Basal secondary: F_(1,19)_ = 2.346, t_(19)_ = 1.532, *P* = 0.142, nested Student’s t-test; Apical oblique: F_(1,19)_ = 0.350, t_(19)_ = 0.592, *P* = 0.561, nested Student’s t-test; Apical tuft: F_(1,19)_ = 0.413, t_(19)_ = 0.643, *P* = 0.528, nested Student’s t-test**;** n = 10 *Csf1r*^+/+^ (4 m/6 f) and 11 *Csf1r*^ΔFIRE/ΔFIRE^ mice (6 m/5 f). **h**) We observed no difference in the number of primary secondary or tertiary dendrites at P28 in CA1 pyramidal cells. Primary: F_(1,20)_ = 0.172, t_(20)_ = 0.415, *P* = 0.682, nested Student’s t-test; Secondary: F_(1,20)_ = 0.909, t_(20)_ = 0.954, *P* = 0.352, nested Student’s t-test; Tertiary: F_(1,20)_ = 2.33, t_(20)_ = 1.53, *P* = 0.143, nested Student’s t-test; n = 11 *Csf1r*^+/+^ (5 m/6 f) and 11 *Csf1r*^ΔFIRE/ΔFIRE^ mice (6 m/5 f).

As shown above, the fractalkine receptor *Cx3cr1* is an established microglial signature gene that is almost entirely absent in *Csf1r*^∆FIRE/∆FIRE^ brains (Extended Data Fig. 1a,b). Mice deficient in CX3CR1 showed transient reductions of microglia P8–P28 in the stratum radiatum of hippocampal area CA1, coinciding with elevated synapse number at P15 as assessed by physical (spine density) and functional analysis (miniature excitatory postsynaptic potential (mEPSC) frequency and amplitude)^3^. The CA1 pyramidal cells (PCs) of *Csf1r*^∆FIRE/∆FIRE^ mice were morphologically similar to wild-type (WT), with dendritic arborization indistinguishable between the two genotypes at P14 (Extended Data Fig. 4a–d) and P28 (Extended Data Fig. 4e–h). We also found no difference in the density of putative dendritic spines on basal, oblique or tuft dendrites of CA1 PCs at P14 or P28 (Fig. 1a,b). We next performed stimulated emission depletion (STED) microscopy to visualize the structure of dendritic spines in *Csf1r*^∆FIRE/∆FIRE^ mice beyond the diffraction limit of light. At P14 in CA1 PCs, we found no genotype-dependent differences in spine overall length, head width or neck length (Fig. 1c,d), and confirmed that spine density was not altered (Fig. 1e). Finally, we confirmed that functional synapses were not impaired by measuring mEPSC frequency or amplitude in CA1 PCs. We found no difference in the quantal strength (mEPSC amplitude) or number (mEPSC frequency) between *Csf1r*^∆FIRE/∆FIRE^ and *Csf1r*^+/+^ mice at either P14 or P28 (Fig. 1f–h). These data show that dendritic spine density and morphology, and synaptic function, were unaffected by an absence of microglia at the developmental stages where differences have been previously reported^3^.

**Fig. 2:**
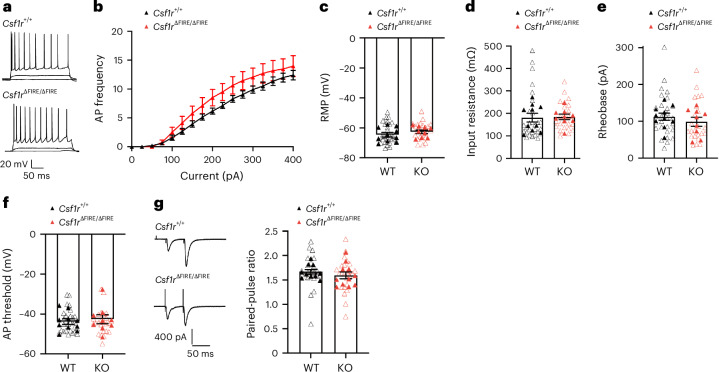
No change in intrinsic neuronal excitability or short-term plasticity in CA1 of the HPC of mice lacking microglia. **a**, Representative traces of the membrane voltage of CA1 PCs at P42 in *Csf1r*^+/+^ (top) and *Csf1r*^ΔFIRE/ΔFIRE^ (bottom) mice in response to hyperpolarizing to depolarizing current steps (−100 to +400 pA, 25-pA steps, 500-ms duration). **b**, AP frequency and current relationship (F/I curve) (*F*_(1,17)_ = 1.60 *P* = 0.223, repeated measures two-way ANOVA (genotype effect), *n* = 10 *Csf1r*^+/+^ mice (seven male mice, three female mice) and *n* = 9 *Csf1r*^ΔFIRE/ΔFIRE^ mice (five male mice, four female mice)). Absence of microglia during early development had no discernible effect on the CA1 pyramidal RMP (**c**, *F*_(1,17)_ = 1.09, *t*_(17)_ = 1.04, *P* = 0.311, nested Student’s *t*-test), input resistance (**d**, *F*_(1,17)_ = 0.032, *t*_(17)_ = 0.178, *P* = 0.861, nested Student’s *t*-test), rheobase (**e**, *F*_(1,17)_ = 1.09, *t*_(17)_ = 1.05, *P* = 0.310, nested Student’s *t*-test) or AP threshold (**f**, *F*_(1,17)_ = 0.045, *t*_(17)_ = 0.212, *P* = 0.834, nested Student’s *t*-test). In **d**–**f**, *n* = 10 (seven male mice, three female mice) *Csf1r*^+/+^ and *n* = 9 (five male mice, four female mice) *Csf1r*^ΔFIRE/ΔFIRE^ mice. **g**, Delivery of paired-pulse electrical stimulation (2× stimuli, 50-ms interval) to the Schaffer collaterals resulted in facilitating EPSCs, which did not differ between genotypes. *F*_(1,19)_ = 0.413, *t*_(19)_ = 0.413 *P* = 0.528, nested Student’s *t*-test (*n* = 10 *Csf1r*^+/+^ mice (six male mice, four female mice) and *n* = 11 *Csf1r*^ΔFIRE/ΔFIRE^ mice (five male mice, six female mice). All data are shown as the mean ± s.e.m.; all statistical tests are two-sided. Source data

**Extended Data Fig. 5:**
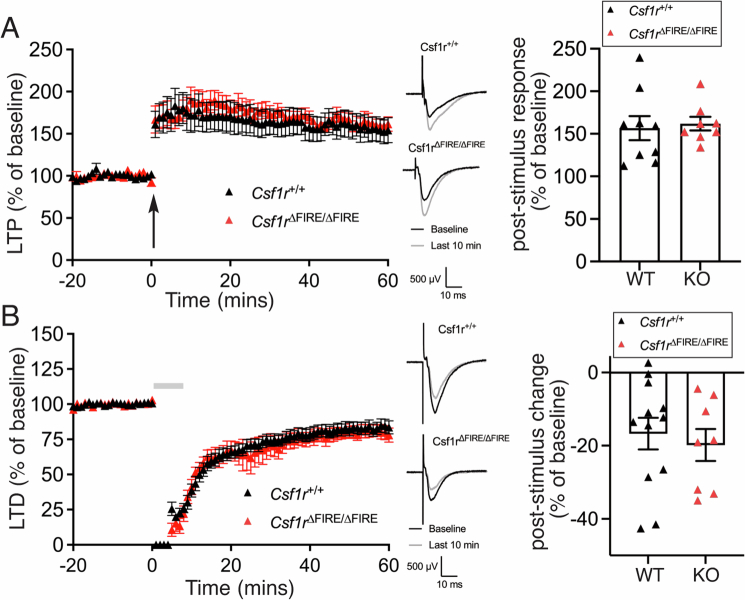
Analysis of synaptic plasticity. All data are shown as mean ± s.e.m. and all statistical tests are two-sided. **a**) Time-course of long-term potentiation (LTP) at Schaffer-Collateral afferents in hippocampal area CA1, for *Csf1r*^+/+^ (black) and *Csf1r*^ΔFIRE/ΔFIRE^ (red) mice at P14. Data is shown for 1 hour following 2×1 s 100 Hz tetanic stimulation (arrow). Example traces are shown for each genotype, before (black) and after (grey) LTP induction. Quantification of the magnitude of LTP for both genotypes revealed no difference (t_(15)_ = 0.31, *P* = 0.761, 2-tailed Student’s t-test), n = 9 *Csf1r*^+/+^ and 8 *Csf1r*^ΔFIRE/ΔFIRE^ mice. **b**) Time-course of long-term depression (LTD) of the Schaffer-Collateral afferents to CA1, induced by application of 50 µM R, S-DHPG (grey bar) in P14 mice. The magnitude of LTD, as measured 50–60 minutes after DHPG application revealed no effect of genotype (t_(18)_ = 0.486, *P* = 0.633, 2-tailed Student’s t-test), n = 12 *Csf1r*^+/+^ and 8 *Csf1r*^ΔFIRE/ΔFIRE^ mice.

In addition to a presumed role in controlling CA1 synapse numbers in early development, microglia have been implicated in regulating the maturation of synapses, that is, in terms of neurotransmitter receptor content and presynaptic function^12^. In putative CA3-CA1 connections measured by stimulating the stratum radiatum in brain slices from *Csf1r*^∆FIRE/∆FIRE^ mice, we observed normal development of the *N*-methyl-d-aspartate receptor (NMDAR):α-amino-3-hydroxy-5-methyl-4-isoxazolepropionic acid receptor (AMPAR) ratio from P14 to P42 (Fig. 1i,j). We also confirmed developmental regulation of NMDAR-mediated EPSC kinetics, a measure of NMDAR subunit composition, finding a temporal reduction in decay time constant to be similar in recordings of CA1 PCs from *Csf1r*^∆FIRE/∆FIRE^ and *Csf1r*^+/+^ mice (Fig. 1k). Consistent with similar synaptic properties in the absence of microglia, we also observed normal synaptic plasticity: induction of long-term potentiation (LTP) and long-term depression (LTD) at Schaffer collateral synapses in *Csf1r*^∆FIRE/∆FIRE^ mice was similar to that of *Csf1r*^+/+^ mice at P14 (Extended Data Fig. 5a,b). In addition to the basal synaptic properties of neurons, how neurons intrinsically respond to synaptic inputs is a critical determinant of brain circuit development. Therefore, we measured the intrinsic physiological properties of *Csf1r*^∆FIRE/∆FIRE^ CA1 pyramidal neurons at the age (P42) studied above. We found intrinsic properties in *Csf1r*^∆FIRE/∆FIRE^ neurons (frequency-current curve, resting membrane potential (RMP), input resistance, rheobase, action potential (AP) threshold), plus paired-pulse ratio, to be indistinguishable from WT (Fig. 2a–g). Thus, microglia are not essential for several aspects of hippocampal CA1 synaptic and cellular development for which they have been proposed to have a role.

### Refinement of eye-specific inputs in the LGN without microglia

**Fig. 3:**
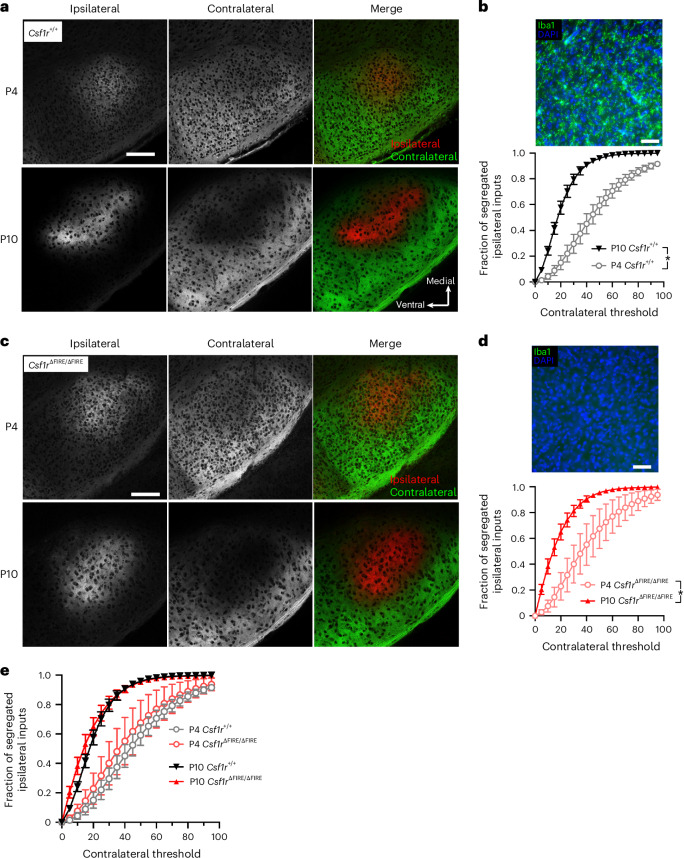
Retinal inputs into the LGN segregate typically in the absence of brain microglia. **a**, Example confocal images of the dorsal LGN of P4 and P10 *Csf1r*^+/+^ mice after anterograde labeling of inputs from the ipsilateral (DiI, red) and contralateral (DiO, green) retinas. **b**, Quantification of dorsal LGN segregation by measuring the fraction of segregated inputs at P4 (open circles) and P10 (closed triangles) in *Csf1r*^+/+^ mice (*F*_(1,12)_ = 46.97, *P = 1.8 × 10^−3^, two-way ANOVA (age effect); *n* = 5 mice (P4, 3 male mice, two female mice), *n* = 9 mice (P10, three male mice, six female mice)). Top, Example image of DAPI/IBA1 staining. **c**, Example images of dorsal LGN segregation in *Csf1r*^ΔFIRE/ΔFIRE^ mice according to the same scheme as **a**. **d**, Dorsal LGN segregation measured at P4 (open circles) and P10 (closed triangles) in *Csf1r*^ΔFIRE/ΔFIRE^ mice (*F*_(1,14)_ = 14.07, *P* = 0.002, two-way ANOVA (age effect)), *n* = 4 (P4, two male mice, two female mice) and *n* = 12 (P10, six male mice, six female mice). Top, Example image of DAPI/IBA1 staining. **e**, Comparison of the dorsal LGN input segregation at P10 (*F*_(1,19)_ = 0.686, *P* = 0.418, two-way ANOVA (genotype)) and P4 (*F*_(1,7)_ = 0.351, *P* = 0.572, two-way ANOVA (genotype)) displayed no difference between genotypes (for *n* see **b** and **d**). All data are shown as the mean ± s.e.m. and all statistical tests are two-sided. **a**, Scale bar, 100 µm. **b**, Scale bar, 50 µm. **c**, Scale bar, 100 µm. **d**, Scale bar, 50 µm. Source data

Besides CX3CR1 signaling, several studies proposed that microglia-mediated synapse removal depends on mechanisms involving components of the complement system, including C1Q (an initiator of the classical complement cascade) and C3 (a target for phagocytosis by CR3-expressing cells such as microglia). This system has been implicated in the axonal refinement of retinal ganglion cells in the LGN, where inputs from both eyes overlap in early development, but later segregate into nonoverlapping eye-specific zones^26–29^. Studies reported that this segregation proceeds normally in *Cx3cr1*^−/−^ mice^30^, but it is impaired in mice deficient in CR3, C3 or C1qa^2,7,8^. *Csf1r*^∆FIRE/∆FIRE^ mice express more than 95% lower *C1qa*, *C1qab* and *C1qac* mRNA in the brain (Extended Data Fig. 1a,b). Consistent with previous studies^26–29^, we observed that inputs into the LGN from both eyes overlap early in development (P4); by P10, they have segregated into eye-specific fields (Fig. 3a,b,e). However, we found that developmental eye-specific segregation in the LGN is normal in *Csf1r*^∆FIRE/∆FIRE^ mice lacking microglia (Fig. 3c–e). Thus, there is no noticeable requirement for microglia for developmental axon terminal refinement in the LGN.

### The barrel cortex of *Csf1r*^∆FIRE/∆FIRE^ mice develops normally

**Fig. 4:**
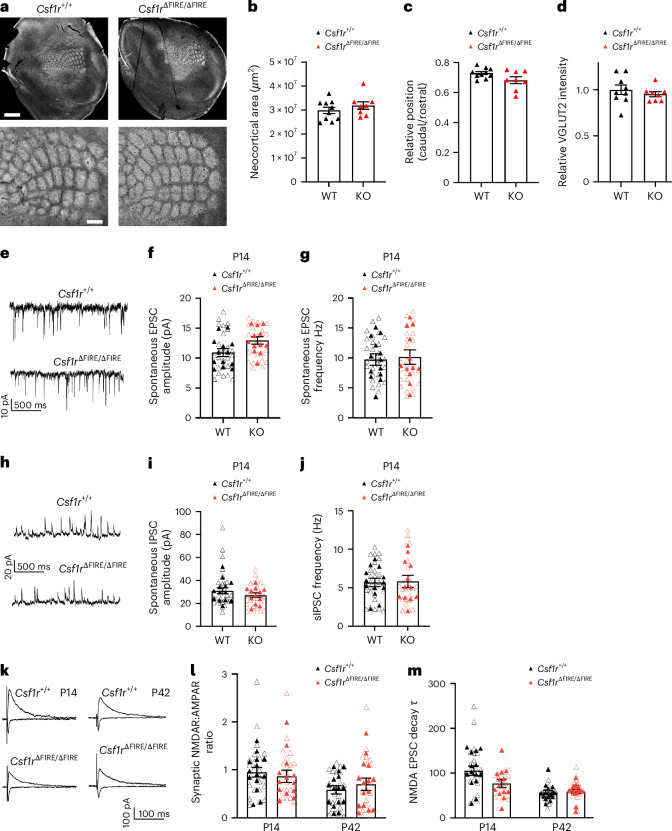
Assessment of synaptic properties in the development of the somatosensory cortex in the absence of microglia. **a**, Example vesicular glutamate transporter 2 (VGLUT2) immunofluorescence images of the somatosensory barrel cortex from *Csf1r*^+/+^ (left) and *Csf1r*^ΔFIRE/ΔFIRE^ (right) mice. **b**, Quantification of the neocortical area occupied by the barrel field (*t*_(16)_ = 1.01, *P* = 0.329, Student’s two-tailed *t*-test), *n* = 10 *Csf1r*^+/+^ and *n* = 8 *Csf1r*^ΔFIRE/ΔFIRE^ mice. **c**, Caudal and rostral relative position of the barrel field (*t*_(16)_ = 2.01, *P* = 0.062, Student’s two-tailed *t*-test), *n* = 10 *Csf1r*^+/+^ and *n* = 8 *Csf1r*^ΔFIRE/ΔFIRE^ mice. **d**, VGLUT2 staining intensity in the barrel cortex: *t*_(15)_ = 0.78, *P* = 0.448, Student’s *t*-test, *n* = 9 *Csf1r*^+/+^ and *n* = 8 *Csf1r*^ΔFIRE/ΔFIRE^ mice. **e**, Example of spontaneous EPSC recordings from P14 L4 stellate cells at −70 mV. **f**,**g**, No difference in spontaneous EPSC amplitude (**f**, *F*_(1,23)_ = 3.36, *t*_(23)_ = 1.83, *P* = 0.081, nested Student’s *t*-test) or frequency (**g**, *F*_(1,23)_ = 0.029, *t*_(23)_ = 0.171, *P* = 0.866, nested Student’s *t*-test), *n* = 13 *Csf1r*^+/+^ mice (eight male mice, five female mice) and *n* = 12 *Csf1r*^ΔFIRE/ΔFIRE^ mice (six male mice, six female mice). **h**, Example of spontaneous IPSC potential recordings from P14 L4 stellate cells. **i**,**j**, No difference in spontaneous IPSC amplitude (**i**, *F*_(1,23)_ = 2.05, *t*_(23)_ = 1.43, *P* = 0.166, nested Student’s *t*-test) or frequency (**j**, *F*_1,23)_ = 0.003, *t*_(23)_ = 0.050, *P* = 0.961, nested Student’s *t*-test); *n* = 13 *Csf1r*^+/+^ mice (eight male mice, five female mice) and *n* = 12 *Csf1r*^ΔFIRE/ΔFIRE^ mice (six male mice, six female mice). **k**, Example of traces of EPSCs recorded in L4 stellate cells. **l**, AMPAR-mediated and NMDAR-mediated EPSCs were measured after stimulation of thalamo-cortical afferents. Quantification of the NMDAR:AMPAR EPSC ratios confirmed no genotype difference at P14 (*F*_(1,25)_ = 0.330, *t*_(25)_ = 0.574, *P* = 0.571, nested Student’s *t*-test) or P42 (*F*_(1,28)_ = 0.809, *t*_(28)_ = 0.899, *P* = 0.376, nested Student’s *t*-test). P14: *n* = 14 *Csf1r*^+/+^ mice (seven male mice, seven female mice) and *n* = 13 *Csf1r*^ΔFIRE/ΔFIRE^ mice (eight male mice, five female mice); P42: *n* = 15 *Csf1r*^+/+^ (eight male mice, seven female mice) and *n* = 15 *Csf1r*^ΔFIRE/ΔFIRE^ mice (six male mice, nine female mice). **m**, Measurement of the NMDAR-mediated EPSC decay time constant (*τ*) revealed a small difference at P14 (*F*_(1,25)_ = 6.69, *t*_(25)_ = 2.59, *P* = 0.016, nested Student’s *t*-test; *n* = 14 *Csf1r*^+/+^ (seven male mice, seven female mice) and *n* = 13 *Csf1r*^ΔFIRE/ΔFIRE^ mice (eight male mice, five female mice)). No difference was observed at P42 (*F*_(1,28)_ = 0.410, *t*_(28)_ = 0.641, *P* = 0.527, nested Student’s *t*-test; *n* = 15 *Csf1r*^+/+^ (eight male mice, seven female mice) and *n* = 15 *Csf1r*^ΔFIRE/ΔFIRE^ mice (six male mice, nine female mice)). All data are shown as the mean ± s.e.m.; all statistical tests are two-sided. **a**, Scale bars, 1 mm (top) and 200 µm (bottom). Source data

**Extended Data Fig. 6:**
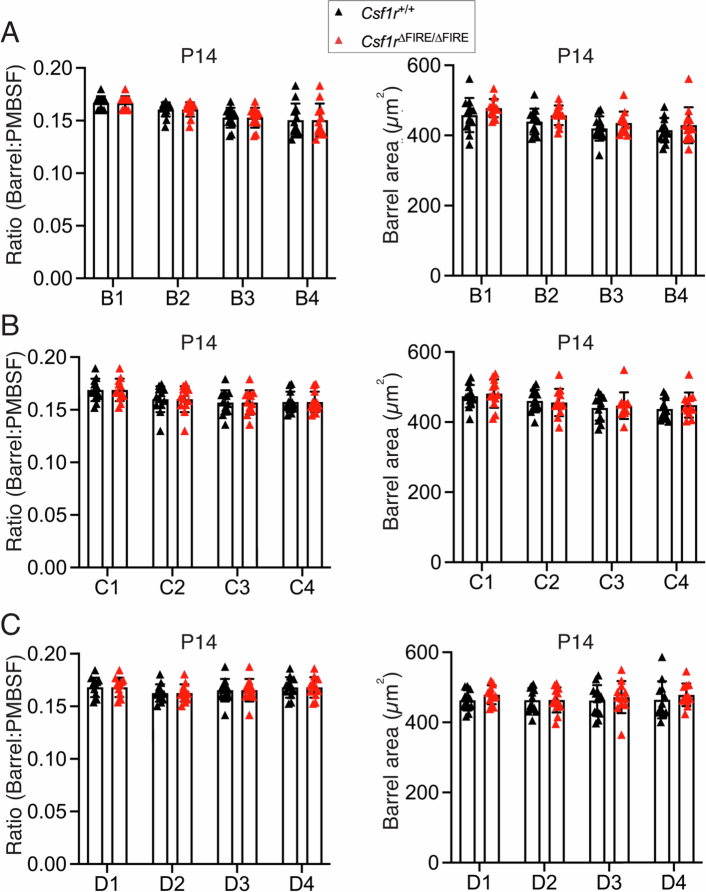
An absence of microglia does not alter the segregation of and size of individual somatosensory barrels. All data are shown as mean ± s.e.m. and all statistical tests are two-sided. Ratio: The ratio of the barrel area in relation to the total area of the PMBSF. Barrel area: area of individual barrels 1–4 in rows B, C and D of the barrel field. **a-c)** Statistics (left-to right): F_(1, 22)_ = 3.26E-31, *P* > 0.999; F_(1, 22)_ = 1.588, *P* = 0.221 (a). F_(1, 22)_ = 1.63E-30, p > 0.999; F _(1, 22)_ = 0.2076, *P* = 0.653 (b). F_(1, 22)_ = 1.165E-29, *P* > 0.999; F_(1, 22)_ = 0.557, *P* = 0.464 (c). All are two-way ANOVAs [genotype effect], n = 12 mice of each genotype.

In addition to the LGN, microglia have also been implicated in the refinement of synapses in the barrel field of the primary somatosensory (S1) cortex^4,10^. The barrel field undergoes stereotypical patterning in layer 4 (L4) between P4 and P10 involving axonal and dendritic refinement. *Csf1r*^∆FIRE/∆FIRE^ mice displayed normal anatomical organization of the barrel field compared to *Csfr1*^+/+^ littermates, both in terms of overall barrel field size but also barrel organization and synaptic labeling (Fig. 4a–d and Extended Data Fig. 6a–c). Next, we determined if basal synaptic transmission was altered in the L4 of S1, as suggested after microglial depletion, after administration of the *CSF1R* inhibitor PLX5622 (ref. ^10^). We found that P14 *Csf1r*^∆FIRE/∆FIRE^ and *Csf1r*^+/+^ L4 S1 neurons had similar spontaneous EPSC and inhibitory postsynaptic current (IPSC) amplitude and frequencies (Fig. 4e–j), leading us to conclude that excitatory and inhibitory synaptic development is not strongly affected by the constitutive absence of microglia.

**Fig. 5:**
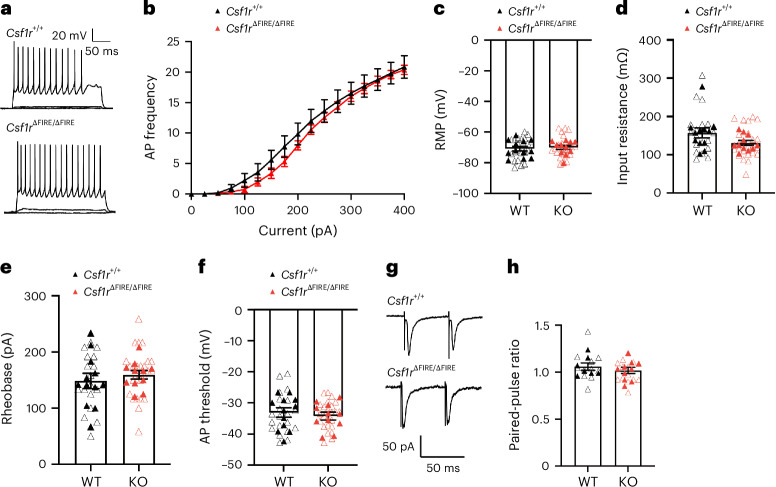
No change in intrinsic neuronal excitability or short-term plasticity in the somatosensory cortex of mice lacking microglia. **a**, Representative traces of the membrane voltage of L4 stellate cells at P42 in *Csf1r*^+/+^ (top) and *Csf1r*^ΔFIRE/ΔFIRE^ (bottom) mice in response to hyperdepolarizing to depolarizing current steps (−100 to +400 pA, 25-pA steps, 500-ms duration). **b**, AP frequency and current relationship (F/I curve) (*F*_(1,21)_ = 0.401 *P* = 0.533, repeated measures two-way ANOVA (genotype effect), *n* = 12 *Csf1r*^+/+^ mice (nine male mice, three female mice) and *n* = 11 *Csf1r*^ΔFIRE/ΔFIRE^ mice (seven male mice, four female mice)). **c**–**f**, We observed no change in RMP (**c**, *F*_(1,21)_ = 0.216, *t*_(21)_ = 0.465, *P* = 0.647, nested Student’s *t*-test), input resistance (**d**, *F*_(1,21)_ = 3.51, *t*_(21)_ = 1.87, *P* = 0.075, nested Student’s *t*-test), rheobase (**e**, *F*_(1,21)_ = 0.766, *t*_(21)_ = 0.875, *P* = 0.391, nested Student’s *t*-test) and AP threshold (**f**, *F*_(1,21)_ = 0.206, *t*_(21)_ = 0.454, *P* = 0.655, nested Student’s *t*-test). In **c**–**f**, *n* = 12 *Csf1r*^+/+^ (nine male mice, three female mice) and *n* = 11 *Csf1r*^ΔFIRE/ΔFIRE^ (seven male mice, four female mice) mice. **g**,**h**, Delivery of paired-pulse electrical stimulation (2× stimuli, 50-ms interval) to thalamo-cortical afferents (**g**) resulted in nonfacilitating EPSCs (**h**), which did not differ between genotypes at P42 (*F*_(1,15)_ = 1.10, *t*_(15)_ = 1.047, *P* = 0.312, nested Student’s *t*-test), *n* = 7 *Csf1r*^+/+^ (five male mice, two female mice) and *n* = 10 *Csf1r*^ΔFIRE/ΔFIRE^ mice (six male mice, four female mice). All data are shown as the mean ± s.e.m.; all statistical tests are two-sided. Source data

We next examined the synaptic AMPAR:NMDAR EPSC ratio and functional NMDAR kinetics in L4 S1 because these properties were reported to be altered in *Cx3cr1*^−/−^ mice^4^. We observed that the AMPAR:NMDAR ratio of S1 L4 neurons was similar in *Csf1r*^∆FIRE/∆FIRE^ and *Csf1r*^+/+^ mice at both P14 and P42 (Fig. 4k,l). Interestingly, the weighted time constant of NMDAR-mediated EPSCs, which reflects the subunit composition of NMDARs, was slightly lower in *Csf1r*^∆FIRE/∆FIRE^ neurons at P14 than WT, although this difference was no longer apparent at P42 (Fig. 4m). Furthermore, the intrinsic physiological properties of L4 neurons (frequency-current curve, RMP, input resistance, rheobase, AP threshold) were indistinguishable from those in *Csf1r*^+/+^ mice, as was the paired-pulse ratio (Fig. 5a–h). Overall, our data demonstrate that the absence of microglia does not compromise normal anatomical or electrophysiological development of the barrel cortex, with the exception of a precociously low decay time constant in NMDAR-mediated synaptic responses at P14.

### Single-nucleus analysis of excitatory and inhibitory neurons

**Fig. 6:**
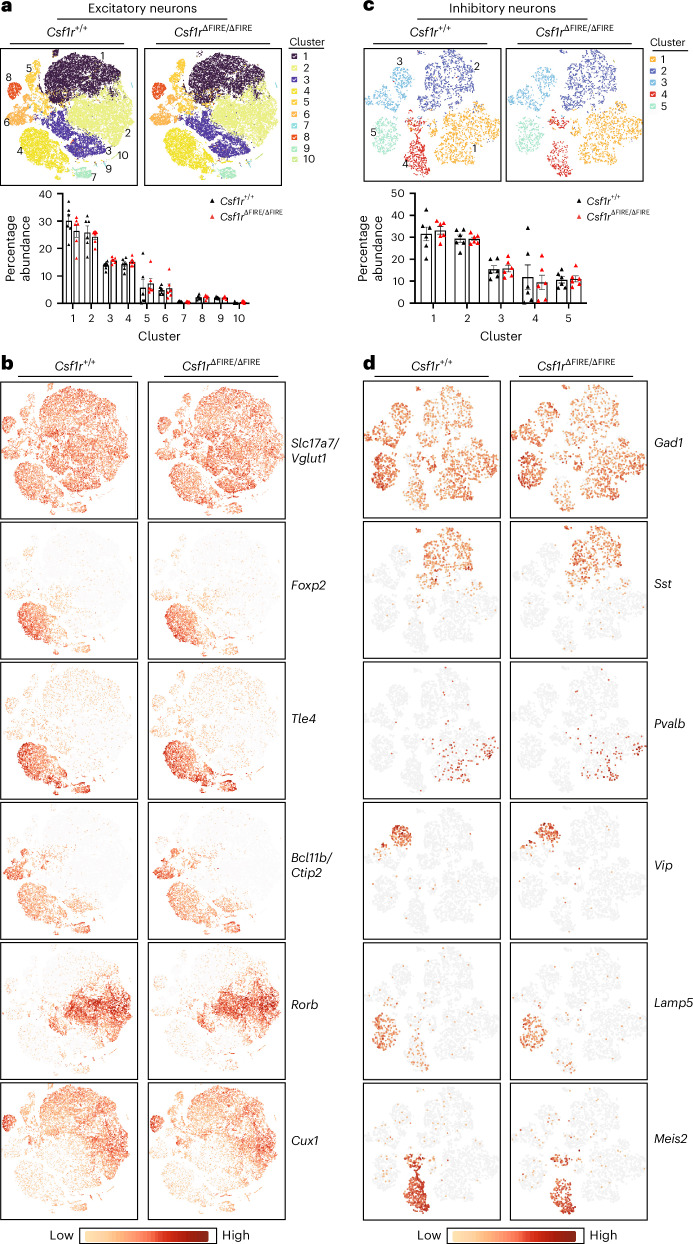
snRNA-seq analysis of neurons from the P14 neocortex. **a**, Clustering of single nuclei (*t*-distributed stochastic neighbor embedding (*t*-SNE) projection) selected based on the expression of the excitatory neuron marker *Slc17a7* (VGLUT1). Top, Clusters of nuclei whose percentage abundance is shown in the bottom graph (*n* = 6 per genotype). *F*_(1,100)_ = 4.3 × 10^−3^, *P* = 0.995 (genotype effect, two-way ANOVA). **b**, The expression of cortical layer markers is compared between genotypes. Pseudobulk differential gene expression analysis revealed no differences in gene expression between *Csf1r*^+/+^ and *Csf1r*^ΔFIRE/ΔFIRE^ mice. **c**, Clustering of single nuclei (*t*-SNE projection) selected based on the expression of the inhibitory neuron markers *Gad1* and *Gad2*. Top, Clusters of nuclei whose percentage abundance is shown in the bottom graph (*n* = 6 per genotype). *F*_(1,50)_ = 0.0004, *P* = 0.985 (genotype effect), two-way ANOVA. **d**, The expression of inhibitory neuron subtype markers was compared between genotypes. Pseudobulk differential gene expression analysis revealed no differences in gene expression between *Csf1r*^+/+^ and *Csf1r*^ΔFIRE/ΔFIRE^ mice. In **a**,**b**, *n* = 6 *Csf1r*^+/+^ mice (three male mice, three female mice) and *n* = 6 *Csf1r*^ΔFIRE/ΔFIRE^ mice (three male mice, three female mice). All data are shown as the mean ± s.e.m.; all statistical tests are two-sided. Source data

To complement our electrophysiological analyses of the developing cortex, we performed single-nucleus RNA-seq (snRNA-seq) on the P14 neocortex to determine whether any changes to the transcriptional profile of excitatory or inhibitory neurons could be detected (Fig. 6). After an initial clustering, we selected clusters expressing the excitatory glutamatergic neuron marker *Slc17a7*, which encodes the vesicular glutamate transporter VGLUT, and reclustered the excitatory neuronal nuclei (Fig. 6a). Excitatory neuronal clusters had similar abundance in *Csf1r*^∆FIRE/∆FIRE^ versus *Csf1r*^+/+^ mice (Fig. 6a). Some clusters expressed several cortical layer markers, including *Foxp2* and *Tle* (both layer VI), *Bcl11b* and *Ctip2* (layers V and VI), *Rorb* (layer IV) and *Cux1* (layers II, III and IV) (Fig. 6b). Moreover, differential gene expression analysis revealed no significantly changed genes (*Csf1r*^∆FIRE/∆FIRE^ versus *Csf1r*^+/+^), including when sex was considered (Supplementary Data 1). We also reclustered inhibitory neurons (clusters identified by the expression of *Gad1* and *Gad2*). We observed that inhibitory neuronal nuclei from *Csf1r*^∆FIRE/∆FIRE^ and *Csf1r*^+/+^ mice clustered together (Fig. 6c), with different clusters enriched in cortical interneuronal subtype markers such as *Sst*, *Pvalb*, *Vip*, *Lamp5* and *Meis2* (Fig. 6d). As with excitatory neurons, we observed no significantly changed genes (*Csf1r*^∆FIRE/∆FIRE^ versus *Csf1r*^+/+^), including when sex was considered (Supplementary Data 2). Thus, we observed no evidence that neuronal transcriptional profiles are substantially altered in the *Csf1r*^∆FIRE/∆FIRE^ neocortex.

### Modest changes to astrocytes observed in *Csf1r*^∆FIRE/∆FIRE^ mice

**Fig. 7:**
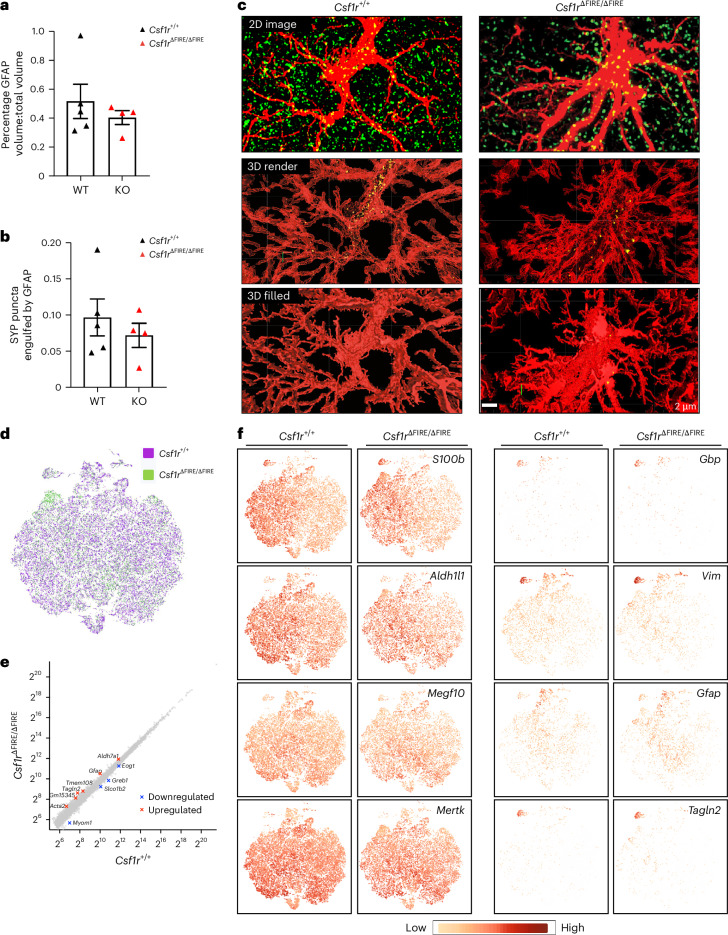
Study of astrocytes in *Csf1r*^ΔFIRE/ΔFIRE^ mice. **a**, Percentage of the total area analyzed occupied by GFAP immunoreactivity in confocal stacks in CA1; *t*_(7)_ = 0.799, *P* = 0.451, unpaired *t*-test (*n* = 5 *Csf1r*^+/+^ mice; *n* = 4 *Csf1r*^ΔFIRE/ΔFIRE^ mice). **b**, SYP puncta (from 10 nm to 1 µm in diameter) engulfed totally by GFAP immunoreactivity (*n* = 5 *Csf1r*^+/+^ mice; *n* = 4 *Csf1r*^ΔFIRE/ΔFIRE^ mice). *t*_(7)_ = 0.760, *P* = 0.472, unpaired *t*-test. **c**, Example images of imaged sections from *Csf1r*^+/+^ and *Csf1r*^ΔFIRE/ΔFIRE^ mice. Top, Raw two-dimensional (2D) maximum projection image. Middle, 3D transparent render with engulfed puncta shown. Bottom, 3D surface render with engulfed puncta now not visible. **d**–**f**, scRNA-seq of astrocytes. **d**, Astrocytes were sorted using FACS from the neocortex at P14 (*n* = 4 per genotype, each two male mice and two female mice) and subject to scRNA-seq (10x Genomics). Small non-astrocyte-contaminating cell populations were removed and astrocytes reclustered. Co-clustering of astrocytes from both genotypes (*t*-SNE projection) is shown. **e**, Results of the pseudobulk gene expression analysis: 11 genes were altered out of 13,257. **f**, Expression of the indicated genes. *S100b* and *Aldh1l1* are astrocyte markers (unchanged). *Megf10* and *Mertk* are key phagocytic genes (unchanged). *Gbp* and *Vim* are reactive markers (unchanged). Examples of altered genes (*Gfap* and *Tagln2*) are shown. All data are shown as the mean ± s.e.m.; all statistical tests are two-sided. **c**, Scale bar, 2 µm. Source data

Astrocytes may also support developmental synapse loss (via MEGF10 and MERTK signaling^31^), raising the possibility that they compensate for an absence of microglia with increased uptake of synaptic material. To investigate this, we studied the presence of the presynaptic marker synaptophysin (SYP) in astrocytes in the CA1 of the HPC at P14, which aligns with our earlier data (Fig. 1) and previous studies^3^. We examined the colocalization of SYP and glial fibrillary acidic protein (GFAP). GFAP was chosen because basal expression is high in the HPC (unlike the neocortex) with strong antibody labeling, acknowledging the caveat that GFAP does not fill the entire cell. Three-dimensional (3D) confocal imaging of GFAP^+^ cells revealed a similar volume occupied by GFAP immunoreactivity in both genotypes (Fig. 7a). While we did observe the presence of SYP puncta in GFAP cell volumes (Fig. 7b,c), the number of engulfed puncta per unit volume of GFAP, including puncta that colocalized with GFAP entirely, was not different between *Csf1r*^∆FIRE/∆FIRE^ and *Csf1r*^+/+^ mice (Fig. 7b). To conclude, while we saw some evidence of astrocytic uptake of synaptic material, we found no evidence of a compensatory upregulation of this in the absence of microglia.

**Extended Data Fig. 7:**
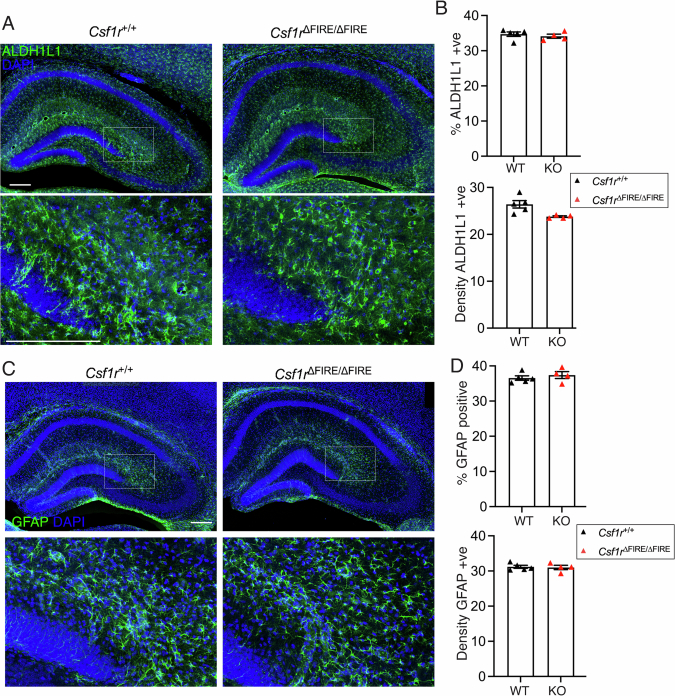
Analysis of astrocytes in the mouse hippocampus. All data are shown as mean ± s.e.m. and all statistical tests are two-sided. **a, b)** Measurement of astrocyte density using an antibody against the pan-astrocyte marker ALDH1L1. Upper graph shows percent of DAPI-positive cells (t_(7)_ = 0.657, *P* = 0.532 (unpaired t-test), lower graph shows density of astrocytes per 100 µm^2^ (t_(7)_ = 2.88, *P* = 0.024 (unpaired t-test)). Scale bar: 200 µm. **c, d)** Analysis as per (a, b) except that an antibody against GFAP was employed (t_(7)_ = 0.750, *P* = 0.478 (upper)); (t_(7)_ = 0.273, *P* = 0.793 (lower, unpaired t-test)), n = 5 *Csf1r*^+/+^ (3 m/2 f) and n = 4 (2 m/2 f) *Csf1r*^ΔFIRE/ΔFIRE^ mice. Scale bar: 200 µm.

**Extended Data Fig. 8:**
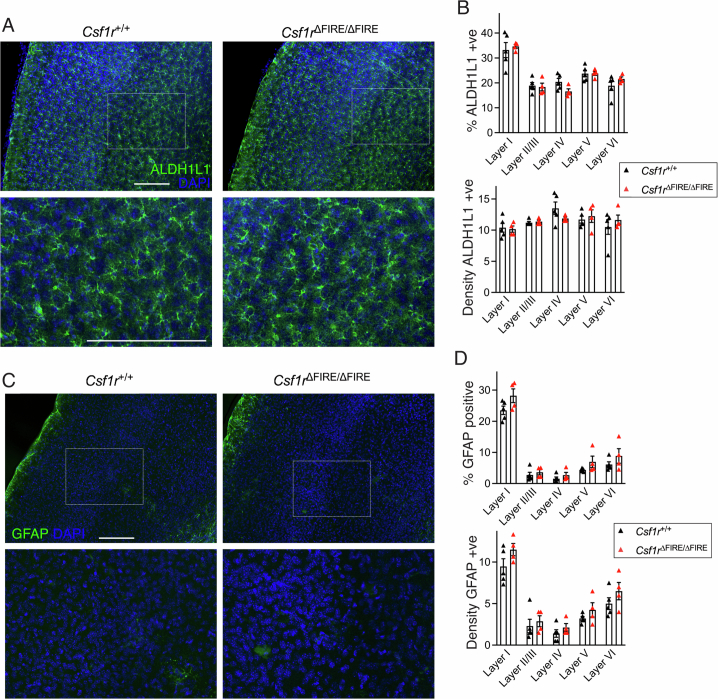
Analysis of astrocytes in the mouse neocortex. All data are shown as mean ± s.e.m. and all statistical tests are two-sided. **a, b**) Measurement of astrocyte density using an antibody against the pan-astrocyte marker ALDH1L1. Upper graph shows percent of DAPI-positive cells; lower graph shows density of astrocytes (per 100 µm^2^). F_(1, 35)_ = 0.0009, *P* = 0.977 (upper); F_(1, 35)_ = 0.1.2E-05, *P* = 0.997 (lower, 2-way ANOVA, genotype effect). Scale bar: 200 µm **c, d)** Analysis as per (a, b) except that an antibody against GFAP was employed. c: F_(1, 35)_ = 8.81, *P* = 0.0054. 2-way ANOVA (genotype effect). No individual layer showed statistical significance (Sidak’s post-hoc test). d: F_(1, 35)_ = 6.55, *P* = 0.015, 2-way ANOVA (genotype effect). No individual layer showed statistical significance (Sidak’s post-hoc test). N = 5 *Csf1r*^+/+^ (3 m/2 f) and n = 4 (2 m/2 f) *Csf1r*^ΔFIRE/ΔFIRE^ mice. Scale bar: 200 µm.

To determine more generally if astrocytes are altered by an absence of microglia, we performed single-cell RNA-seq (scRNA-seq) on fluorescence-activated cell sorting (FACS)-sorted cortical astrocytes at P14 of *Csf1r*^∆FIRE/∆FIRE^ and *Csf1r*^+/+^ mice (*n* = 4 per genotype, sex-balanced). Astrocytes from *Csf1r*^∆FIRE/∆FIRE^ and *Csf1r*^+/+^ mice consistently co-clustered together (Fig. 7d); both genotypes displayed a small reactive-like cluster (enriched in the reactive markers *Vim* and *Gbp2*; Fig. 7f). Differential gene expression analysis revealed only ten genes to be altered, with only one (*Myom*) changed twofold or more out of 13,257 genes meeting the expression level cutoff (Fig. 7e). A subtle increase in *Gfap* was observed as well as the reactive astrocyte-associated gene *Tagln2*, but no evidence of global astrocyte reactivity at this developmental stage (Fig. 7e,f). Examination of GFAP immunoreactivity revealed a small increase in the cortex but not HPC (Extended Data Figs. 7c,d and 8c,d). Thus, the astrocyte transcriptome exhibited quite subtle changes in *Csf1r*^∆FIRE/∆FIRE^ mice, although this does not rule out that they may compensate independently of changes in gene expression.

**Fig. 8:**
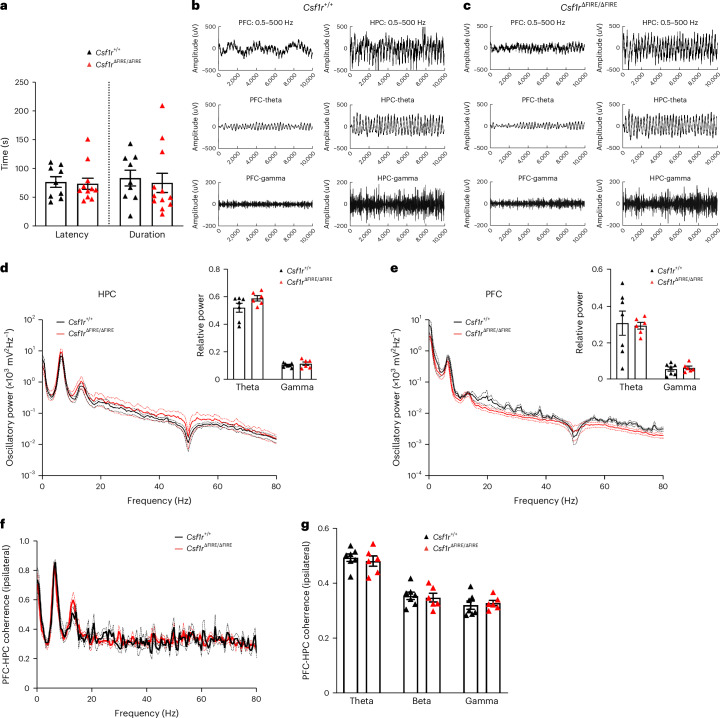
Normal seizure susceptibility and coherence of HPC and PFC oscillatory activity in awake behaving animals. **a**, After administration of PTZ, we compared seizure latency (*t*_(18)_ = 0.221, *P* = 0.828, two-tailed Student’s *t*-test, *P* = 0.882 Mann–Whitney *U*-test) and total seizure duration (*t*_(18)_ = 0.233, *P* = 0.819 two-tailed Student’s *t*-test, *P* = 0.565 Mann–Whitney *U*-test) between genotypes; *n* = 9 *Csf1r*^+/+^ and *n* = 11 *Csf1r*^ΔFIRE/ΔFIRE^ mice. **b**,**c**, Example traces of LFPs from HPC and PFC of the indicated genotypes (*Csf1r*^+/+^ (**b**) and *Csf1r*^ΔFIRE/ΔFIRE^ (**c**)) subjected to a band-pass filter. **d**,**e**, Spectral plot of oscillatory power of LFP in the CA1 of HPC (**d**) or PFC (**e**) of *Csf1r*^+/+^ (black) and *Csf1r*^ΔFIRE/ΔFIRE^ (red) mice. The reduction in power around 50 Hz reflects notch filtering of the electrical line frequency. Insets, Quantification of relative oscillatory power for the theta (4–12 Hz) and gamma (30–80 Hz) frequency bands, normalized to total power. We observed no difference in relative power between genotypes in the HPC (*F*_(1,22)_ = 3.57, *P* = 0.072, two-way ANOVA) or PFC (*F*_(1,22)_ = 0.009, *P* = 0.926, two-way ANOVA (genotype)); *n* = 7 *Csf1r*^+/+^ (three male mice, four female mice) and *n* = 6 *Csf1r*^ΔFIRE/ΔFIRE^ mice (three male mice, three female mice). **f**,**g**, In vivo recording of LFPs in both CA1 of the HPC and of the medial PFC (**f**) revealed no apparent divergence in oscillatory coherence in theta, beta or gamma bands (**g**). *F*_(1,33)_ = 0.117, *P* = 0.735, two-way ANOVA (genotype effect); *F*_(2,33)_ = 0.249, *P* = 0.781 (genotype-frequency interaction). All data are shown as the mean ± s.e.m.; all statistical tests are two-sided. Source data

### Normal seizure susceptibility and long-range activity coherence

Previous reports linking impaired microglial function and hippocampal synapse number in *Cx3cr1*^−/−^ mice also reported reduced susceptibility to the proconvulsant drug pentylene tetrazole (PTZ), attributed to a delay in microglia-dependent brain-wide circuit development^3^. Given that development of synaptic and neuronal properties are apparently normal in *Csf1r*^∆FIRE/∆FIRE^ mice, we wanted to determine whether seizure susceptibility was affected. We detected no difference in seizure latency and duration in *Csf1r*^∆FIRE/∆FIRE^ versus *Csf1r*^+/+^ mice when PTZ seizures were elicited under the same conditions (Fig. 8a).

Beyond simple seizure vulnerability, neuron-microglia signaling has been proposed to have a role in controlling long-range functional brain connectivity: young adult *Cx3cr1*^−/−^ mice displayed a reduced coherence of local field potentials (LFPs) between the medial prefrontal cortex (PFC) and the ipsilateral HPC when awake and exploring^32^. We performed a similar analysis of HPC and medial PFC LFPs in *Csf1r*^∆FIRE/∆FIRE^ versus *Csf1r*^+/+^ in awake mice when exploring a virtual environment, at a similar age as previously reported^32^. We performed spectral power analysis of LFPs recorded from the HPC and ipsilateral medial PFC (Fig. 8b–e), and consequently assessed PFC-HPC coherence (Fig. 8f,g). We found that medial PFC-HPC coherence was not different in *Csfr1*^+/+^ versus *Csf1r*^∆FIRE/∆FIRE^ mice (Fig. 8f,g), suggesting that long-range connections are established normally in the absence of microglia.

## Discussion

Overall, we conclude that developing mice lacking microglia show remarkably normal synaptic density, maturation and patterning in regions where a role for these cells has been previously proposed. This conclusion is not necessarily incompatible with the previous literature reporting phenotypes of mice deficient in microglia-enriched genes such as *Cx3cr1* and *Trem2*. Rather than losing microglial function, mice deficient in *Cx3cr1* or *Trem2* may be phenotypically different due to microglia acquiring aberrant properties. For example, *Cx3cr1-*deficient microglia are morphologically different, have altered electrophysiological properties and respond abnormally to ATP stimuli^33^. *Trem2*-deficient microglia also have altered morphology and responses^13,34^. It is possible that *Cx3cr1*-deficient or *Trem2-*deficient microglia have a gain-of-function effect that is responsible for the phenotypes observed. It is also possible that the presence of dysfunctional microglia in some way impairs the ability of the developing brain to adapt in the way that it potentially does successfully in the *Csf1r*^∆FIRE/∆FIRE^ mouse. However, some aspects of brain development have an absolute requirement for microglia. A recent study showed that microglia have a (largely prenatal) role in closing cortical boundaries, and that *Csf1r*^∆FIRE/∆FIRE^ mice exhibited a cavity at the cortico-striato-amygdalar boundary that extended to P7 (ref. ^35^), confirming that any compensation or adaptation to microglial absence does not result in a totally WT phenotype for developing *Csf1r*^∆FIRE/∆FIRE^ mice. Nevertheless, it is possible that in the context of control of synapse numbers and patterning, that astrocytes carry out functions that ordinarily are carried out by microglia. It is potentially interesting that astrocytes in *Csf1r*^∆FIRE/∆FIRE^ mice showed a tendency to increased reactivity: we observed a small increase in GFAP expression at P14 (Fig. 7e and Extended Data Fig. 8); by P42 our bulk RNA-seq data also showed an increase in reactive markers, including *Gfap*, *Vim* and *Serpina3n*, suggesting that reactivity may increase as mice age. Whether microglia directly signal to astrocytes to prevent reactive astrogliosis is a topic for future investigation.

Another possible explanation for our findings is that the developmental functions previously attributed to microglia were based on studying mice globally deficient in *Cx3cr1*, *Cr3*, *C3* or *C1qa*^3,4,6–8,36–38^, meaning that nonmicroglial processes may contribute to their phenotype. For example, CX3CR1 and the complement system are implicated in the regulation of energy metabolism, angiogenesis and blood–brain barrier integrity^15–19^, which may indirectly affect synaptic turnover. Moreover, complement is known to perform an increasing number of roles in the brain, including neural precursor proliferation and radial migration^20–22^. Greater use of KO models that are cell-type-specific and inducible will help to attribute gene function more precisely to cell type and developmental stage.

A recent study used the *Csf1r*^∆FIRE/∆FIRE^ mouse to study aspects of neuronal development, focusing on the HPC^39^. Consistent with our observations, they did not observe any change in spine density or miniature EPSC frequency; however, they observed a reduction in excitability, reporting that the rheobase was higher in *Csf1r*^∆FIRE/∆FIRE^ neurons. This is a surprising finding from a biophysical point of view in the absence of a change to either AP threshold, RMP or input resistance. One difference between the studies is that the rheobase recorded by Surala et al.^39^ is calculated using large current steps (25 pA) relative to the rheobase (50 pA), yielding highly categorical data, whereas we used a linear ramp for the CA1 pyramidal cells. The authors also reported a reduction in synaptic NMDAR-mediated charge transfer, which we do not observe. The authors calculated the NMDA component of a miniature EPSC, rather than the usual approach of measuring AP-evoked responses (Fig. 1j). Further studies are needed to rationalize these conflicting results.

Another recent study studied the role of microglia in experience-dependent maturation and function of visual circuitry^40^. The authors used the CSF1R antagonist PLX5622 to kill microglia from P14 and found no effect on visual signaling, neuronal tuning properties in the visual cortex and ocular dominance plasticity. The absence of a role for microglia in cortical plasticity resembles our observation in the hippocampal CA1 region (Extended Data Fig. 5) and is consistent with a previous report demonstrating that experience-dependent plasticity in the visual cortex is unchanged in Cx3CR1-deficient mice^30^.

To summarize, the widely used term synaptic pruning implies that microglia have an active function as ‘gardeners’^3^ in removing specific synapses to refine neurological circuits. This and other aspects of synaptic maturation require microglia to appropriately set neural circuits and connectivity. Our study demonstrates that multiple aspects of neuronal development and synapse maturation and refinement can proceed normally when microglia are absent, revealing the adaptability of brain development to the absence of this cell type.

## Methods

### Animals

All experiments were performed under licenses approved by the UK Home Office according to the Animals (Scientific Procedures) Act, having been approved by the University of Edinburgh Local Ethical Review Board. This study used *Csf1r*^∆FIRE/∆FIRE^ mice and control *Csf1r*^+/+^ animals, which were generated as littermates by heterozygous *Csf1r*^∆FIRE/+^ inter-crossing. The creation and characterization of the *Csf1r*^∆FIRE/∆FIRE^ mice is described elsewhere^23^. Mice were on a mixed background: 64–68% homozygous for C57BL/6J, 22–25% CBA and 7–11% heterozygous, with approximately 2% unattributable (Mini Mouse Universal Genotyping Array). Animals were housed in communal cages (maximum six per cage) with ad libitum water and food in a normal 12 h light–dark cycle. Both male and female mice were used throughout the study. Experimenter and data analyzer were blind to the genotype. Sample size was estimated using 3R (reduction, replacement and refinement) principles, with sample size numbers calculated to achieve satisfactory power based on the variance and effect size in published data^3,4,6–8,10,12,38^.

### Mouse pup innate motor tasks

Mice aged P3–P10 underwent two behavioral experiments to assess motor function: (1) righting reflex and (2) negative geotaxis. Each mouse completed both the righting reflex and negative geotaxis tasks every day. In between the tasks, pups were returned to the heated cage to rest. When the session was over, pups were placed in their home cage and the dam was observed for signs of stress. Mice pups spent a maximum of 1 h separated from the dam.

#### Innate righting reflex task (P3–P7)

Mice were placed on their backs on a soft platform, with their paws held together for 3 s. Each mouse was released and the time taken for the pup to return to the prone position was recorded. A trial was defined as successful if mice righted themselves with all four paws on the floor. Mice were given three 15-s trials with a 20-s interval for recovery. All mice successfully managed to right themselves within the given time.

#### Negative geotaxis task (P3–P10)

This is the innate ability of rodents to recognize their orientation on a slope and to turn around so they are facing uphill. Mice were placed head pointing downwards on a 30-degree incline. Mice were placed so that all four paws were touching the surface of the incline. Each pup was released and the time taken for it to turn uphill was recorded. A hand was placed approximately 10 cm below the mouse to catch it if it fell. If a mouse fell, it was replaced back on the slope in the starting position and given another trial. This was repeated until the mouse successfully reorientated itself or until a maximum of ten attempts (nine falls) were made or until 90 s had passed. If the pup showed signs of stress, such as urination or defecation, the trial ended early. The number of failed attempts and the duration to success were recorded.

### IHC

Mice were anesthetized with sodium pentobarbital and perfused transcardially with ice-cold PBS followed by 4% paraformaldehyde (PFA) in PBS. Brains were removed from the skull and postfixed in 4% PFA for 24 h. Brains were cryo-protected in 30% sucrose overnight and then mounted on a freezing microtome in optimal cutting temperature compound, where coronal 50-µm brain sections were prepared. Brain slices were placed in a well plate and washed with 0.1 M phosphate buffer and PBS. Slices were then blocked from unspecific binding using 10% normal goat serum (NGS), 0.3% Triton X-100 and 0.05% Na azide and PBS for 1 h at room temperature. Then, the primary antibody solution containing 10% NGS, 0.3% Triton X-100 and 0.05% Na azide and PBS was added (1:1,000 dilution Iba1, cat. no. ab283319, Abcam; 1:500 dilution Iba1, cat. no. 019-019741, Wako; 1:1,000 dilution GFAP, cat. no. CH22102, Neuromics; 1:1,000 dilution Aldh1l1, cat. no. 702573, Invitrogen; 1:500 dilution SYP, cat. no. 102-002, Synaptic Systems). Slices were incubated for 1 h at room temperature followed by 24 h at 4 °C. The well plate was removed and allowed to come to temperature before the secondary antibody solution was added (10% NGS, 0.3% Triton X-100, 0.05% Na azide and PBS) for 24 h. Then, slices were washed with PBS and 0.1 M phosphate buffer for 1 h before being mounted on glass slides and cover slipped. Images were obtained using a Leica SP8 confocal microscope. 1,024 × 1,024 pixel images of the CA1 (Bregma anteroposterior: −1.75 to −1.9 mm) and the dorsal LGN (Bregma anteroposterior: −2.25 to −2.5 mm) were taken using the ×10 (numerical aperture (NA) = 0.45) and ×20 (NA = 0.8) objectives. Multiple images were taken for each animal. To analyze astrocyte density, images were opened using the ImageJ software. For cell counts, a counting grid consisting of 200 × 200 µm squares was superimposed over the stratum radiatum of the CA1. Using the cell counter tool, the number of 4′,6-diamidino-2-phenylindole (DAPI)^+^, GFAP^+^ and ALDH1L1^+^ cells were quantified. Each DAPI, GFAP and ALDH1L1 cell was counted only if the cell body was within the grid and over 50% of the protrusions were within the grid. The average cell density was measured for each animal and reported as the number of GFAP and ALDH1L1 per 100 µm^2^ and as the percentage of total DAPI cells.

### Bulk, sc-RNA-seq and snRNA-seq

Bulk tissue RNA extraction was carried out as described in ref. ^23^. Briefly, brains from saline-perfused mice were dissected, snap-frozen and subsequently disrupted in the Precellys 24 Homogenizer (Bertin Instruments). RNA isolation was performed using the RNeasy Plus Mini Kit (QIAGEN). RNA-seq reads were mapped to the primary assembly of the mouse reference genome using the STAR RNA-seq aligner v.2.7.11 (ref. ^41^). Tables of per-gene read counts were generated from the mapped reads using featureCounts^42^. Differential gene expression was then performed in R using DESeq2 (ref. ^43^). For bulk RNA-seq, 6–7 mice per genotype were analyzed. To sort astrocytes (4–5 mice per genotype), mouse neocortices were collected then dissociated with Adult Brain Dissociation Kit (Miltenyi Biotec) on a gentleMACS Octo Dissociator using program 37C_ABDK_01. Dissociated samples were then treated with debris and red blood cell removal steps to obtain cell suspensions. For FACS, cells were resuspended to a final volume of 100 μl 0.1% phosphate buffer and incubated with fluorescence-conjugated antibodies at 4 °C for 30 min. The following antibodies were used: ACSA2 APC (1:200 dilution, cat. no. 130-116-245, Miltenyi Biotec) and O4 PE (1:100 dilution, 30-117-357, Miltenyi Biotec). ACSA2 APC^+^ and O4-PE^−^ live single cells were selected as the astrocyte population. These were then loaded onto a 10x Chromium Controller. For snRNA-seq, neocortices were acutely collected, flash-frozen and then stored at −80 °C. A nucleus isolation kit (cat. no. NUC201-1KT, Sigma-Aldrich), dithiothreitol (DTT) (cat. no. R0861), RNase inhibitor (cat. no. AM2694 Thermo Fisher Scientific), 30 µm cell strainer (Partec CellTrics) and 30-G insulin needles (cat. no. 324826, BD) were used. Cryopreserved tissue samples were thawed on ice. Lysis was performed with PURE buffer, 0.1 M DTT, 10% Triton X-100 and RNase inhibitor. The samples were mechanically dissociated in lysis buffer using a P-1000 pipette ten times, and a further three times with insulin needles. The lysate was mixed with a sucrose cushion, which was prepared using PURE 2M sucrose, sucrose cushion buffer, 0.1 M DTT and RNase inhibitor. This mixture was then filtered through a 30-µm cell strainer. In a new low-binding tube, 200 µl of sucrose cushion was added and carefully overlaid with 560 µl of the filtrate. The samples were centrifuged for 45 min at 4 °C. The supernatant was discarded and the pellet was resuspended in Dulbecco’s PBS (DPBS) (cat. no. 14190-094) + 0.5% BSA with RNase inhibitor. The suspension was then centrifuged at 1,000*g*, repeated and finally resuspended in 200 µl Dulbecco’s PBS + 0.5% BSA with RNase. Sorting of nuclei (six mice per genotype) was done using flow cytometry. Gating was set based on size and granularity using forward and side scatter to capture singlets and remove debris. Nuclei were stained with DAPI for detection. Quality control was conducted using Luna FX7 with Acridine Orange/Propidium Iodide; and the input nuclei number was 20,000 per sample. For library preparation, nuclear suspensions were loaded onto a 10x Chromium Controller (10x Genomics). Single-nucleus transcriptomic amplification and library preparation were conducted with the Chromium Single Cell 3′ v.3.1 Reagent Kit (10x Genomics). Libraries were first sequenced on the iSeq 100 System (cat. no. 20021532, Illumina) using the iSeq 100 i1 Reagent v.2 (300 cycle) Kit (cat. no. 20031371). Library molarity for sequencing was calculated using the Qubit double-stranded DNA quantification results and the fragment size information from the Bioanalyzer results. Libraries were normalized to 10 nM and equal volumes were pooled and diluted for sequencing. The PhiX Control v.3 (cat. no. FC-110-3001) library was spiked into the run at a concentration of ~4% to help with cluster resolution and facilitate troubleshooting. iSeq data were then analyzed so that pools could be rebalanced for subsequent deep sequencing. Deep sequencing was performed on the NextSeq 2000 platform (cat. no. SY-415-1002, Illumina) using the NextSeq 1000/2000 P3 Reagents (100 cycles) v.3 Kit (cat. no. 20040559). The PhiX Control v.3 library was spiked in at a concentration of ~1%. For single-cell and single-nucleus sequencing data, sequencing reads were mapped to the mouse genome; per-cell, per-gene count matrices were produced using 10x CellRanger v.7.0.1 (ref. ^44^). Quality control, normalization and clustering of data were performed using the Seurat R package, v.4.4.0 (ref. ^45^). For snRNA-seq, ambient RNA was estimated and removed using the SoupX R package v.1.6.2 (ref. ^46^). Doublets were identified and removed using the scDblFinder R package v.1.10.1 (ref. ^47^). Pseudobulk differential expression analysis was performed by summarizing single-cell gene expression profiles at the individual level using the aggregateBioVar R package v.1.6.0 (ref. ^48^); then differentially expressed genes between genotypes were calculated using DESeq2 v.1.36.0.

### Slice preparation and patch-clamp and field potential recordings

Acute slices were prepared as described previously^49^. Once cut, slices were transferred to a holding chamber containing carbonated sucrose-artificial cerebrospinal fluid (CSF) (whole-cell recordings) or aCSF (field recordings). Slices were allowed to recover for 30 min or longer at 35 °C until needed. For the electrophysiological recordings, slices were transferred to a submerged recording chamber, perfused with carbonated aCSF^49^ at a flow rate of 3–6 ml min^−1^ at 30–31 °C.

For the whole-cell patch-clamp recordings, slices were placed in the recording chamber of an upright microscope (SliceScope, Scientifica) and visualized using infrared differential interference contrast microscopy. For the CA1 recordings, a stimulating electrode was placed in the stratum radiatum. Cells were chosen under high magnification in the stratum pyramidale as having large ovoid somata, with a clear apical dendrite entering the stratum radiatum. For L4 of the primary S1 cortex, stimulating electrodes were placed in either the ventrobasal thalamus or the internal capsule. L4 was identified based on the presence of barrel-like structures observed under low magnification. For the whole-cell recordings, borosilicate glass microelectrodes were pulled on a horizontal electrode puller (model P-87 or P1000, Sutter Instruments), which was filled with either Cs-gluconate-based (140 mM Cs-gluconate, 4 mM CsCl, 0.2 mM EGTA, 10 mM HEPES, 2 mM MgATP, 2 mM Na_2_ ATP, 0.3 mM Na_2_-GTP, 10 mM Na_2_-phosphocreatine, 2.7 mM biocytin and 5 mM QX-314, pH 7.4, 290–310 mOsm) or a K-gluconate-based (142 mM K-gluconate, 4 mM KCl, 0.5 mM EGTA, 10 mM HEPES, 2 mM MgCl_2_, 2 mM Na_2_ ATP, 0.3 mM Na_2_ GTP, 10 mM Na_2_ phosphocreatine and 2.7 mM biocytin, pH 7.4, 290–310 mOsm) internal solutions, which gave a tip-resistance of 5-6 mΩ. Signals were recorded using a Multiclamp 700B amplifier, digitized using a Digidata 1550b (10 or 20 kHz). Cells were rejected if they required a holding current greater than 200 pA to maintain the voltage clamp at −70 mV, if series resistance started at more than 30 mΩ, or if series resistance changed by more than 20% over the course of the recording. Miniature and evoked synaptic recordings were carried out after wash-in of the Cs-gluconate internal solution for 2–5 min at a holding potential of −70 mV. Miniature EPSCs were recorded in the presence of 300 nM tetrodotoxin. To measure the NMDA:AMPA ratio, CA1 pyramidal neurons or S1 L4 neurons were recorded in the presence of 50 µM picrotoxin. Evoked PSCs were generated using a twisted Ni:Chrome bipolar wire connected to either a constant-voltage or constant-current stimulator (Digitimer). In hippocampal slices, the stimulus intensity was adjusted as to produce a monosynaptic AMPAR-mediated EPSC of approximately 200 pA. For the S1 recordings, an extracellular field electrode (a patch pipette filled with aCSF) was first placed in an L4 barrel; electrical stimuli were delivered to identify the synaptic connections. The stimulating electrode was moved between consecutive barrels until a synaptic response was identified. After identifying connectivity, neurons were then recorded in that barrel. Monosynaptic AMPAR-mediated EPSCs were generated (WT_median_ = −86 pA, range: −17 to −491 pA; *Csf1*^+/FIRE^_median_ = −68 pA, range: −8 to −346 pA). EPSC amplitudes were measured as the average peak amplitude (over a 2-ms average) of ten responses, elicited 20 s apart. Assessment of NMDAR function was performed at +40 mV. For mixed AMPA and NMDA EPSCs, the mean response 50–60 ms after stimulus onset, where the AMPAR response had completed its decay, was taken as a proxy of NMDAR response amplitude. For GABA_A_ receptor-mediated IPSCs, neurons were held at 0 mV in a voltage clamp.

The intrinsic properties of neurons were measured using a K-gluconate internal solution. The RMP of the cell was recorded with current clamped at 0 pA; all other protocols were recorded with the appropriate current injection to hold the cell at −70 mV. Input resistance and the membrane time constant were calculated by injecting a − 10 pA step. Current-frequency responses were assessed using a series of 500-ms rectangular current injections ranging from 25–400 pA (25-pA steps). Rheobase in CA1 neurons was assessed using a linear ramp from −100 to 400 pA over the course of 2 s. Analysis of the whole-cell recordings was performed using the open-source software packages Stimfit and Clampfit, or custom-written MATLAB scripts (Supplementary Software 1 and 2). After recording, neurons were resealed by generating outside-out patches, then fixed with 4% PFA for subsequent visualization and IHC.

For hippocampal field EPSP (fEPSP) recording, the CA3 region was removed. Then, fEPSPs were elicited by delivering a short pulse of electrical current (0.1 ms) to Schaffer collateral axons through a twisted Ni:Chrome bipolar wire connected to either a constant-voltage or a constant-current stimulator. Borosilicate glass recording microelectrodes with a resistance ranging from 1 to 3 mΩ were pulled and filled with aCSF. Stimulus intensity was set to 50% of the maximum fEPSP amplitude. LTP was induced by high frequency (two trains of 1-s 100-Hz stimulation, 20 s intertrain interval) after 20 min or more of stable baseline. Data were amplified through an EXT-02B amplifier (NPI Electronic) and digitized at a rate of 20 kHz through a BNC-2090A terminal block (National Instruments). Data acquisition and analysis were performed on WinLTP^50^.

### STED microscopy

Hippocampal slices from P28 mice were prepared for slice physiology; individual CA1 PCs were filled with biocytin using a patch. Slices were incubated at 4 °C overnight in solution containing streptavidin conjugated to Abberior STAR 580 (1:500, cat. no. ST580), 3% NGS, 0.1% Triton X-100 and 0.05% Na azide. Slices were washed with 0.1 M phosphate buffer, mounted onto glass slides. Time-gated STED images were obtained using a STED microscope (Leica SP8 ×3 time-gated STED). Individual apical oblique dendrites from CA1 PCs were imaged using a high-magnification objective (×93 glycerol immersion objective lens, NA = 1.3). Sections were illuminated with a 580-nm light. Image stacks of apical oblique dendritic sections in the stratum radiatum of the CA1 were imaged using a step size of 0.09 µm. The image stacks were Nyquist-sampled with a pixel size of 20 nm and at a scan speed of 200 Hz using six-line averaging. STED images were deconvolved using Huygens STED module (Scientific Volume Imaging). Measurement of the spine morphological parameters were carried out using ImageJ. For WT animals, 124 spines from five animals were measured. For KO animals, 138 spines from five animals were measured. Two dendritic sections were imaged per cell and 1–4 cells were imaged per animal. Statistics were performed on the number of independent biological replicates (that is, animals).

### Labeling, imaging and analysis of contralateral and ipsilateral retinal inputs into the dorsal LGN

P4–5 and P10–11 mice were anesthetized with isoflurane and then decapitated. The skin was removed from the head and the snout clipped. The cornea was peeled back to expose the inner part of the eye. Humorous jelly was removed exposing the retina and optic nerve. Crystals of the lipophilic dye (DiD, cat. no. D7757, Thermo Fisher Scientific, or DiI, cat. no. 60010, Biotium) were placed inside the eye to differentially label the contralateral and ipsilateral eye; the cornea was replaced. The head was stored in 4% PFA and incubated at 37 °C for a week. PFA was then removed and replaced with PBS and 0.1% Na azide and incubated at 37 °C for a further 8 weeks.

After incubation, the head was removed and placed in 4% PFA overnight at 4 °C. The brain was removed from the skull and 50-µm slices were prepared and mounted. Images of the dorsal LGN were obtained using a Zeiss LS800 confocal microscope using the ×20 objective (NA = 0.8), 1024 × 1024 pixels and tiled together. All images were acquired and analyzed blind to genotype (Bregma anteroposterior: −2.25 to −2.5 mm). Image analysis was performed using the Fiji package of ImageJ^51^. The ventral region of the LGN receives no contralateral input from the retina; as such, the ventral LGN should have a fluorescence level similar to that of background. If images failed to meet this criterion they were omitted from the analysis. Images were opened in ImageJ and split into two channels (ipsilateral and contralateral). Background fluorescence was removed using the rolling ball radius filter, set to a diameter of 200 pixels. The outline of the dorsal LGN was drawn using the freehand tool; this excluded the ventral LGN and the optic tract. This outline was saved using the region of interest (ROI) manager tool. The threshold of the contralateral region was set for multi-threshold levels, 0–150 (in 5-pixel steps). A mask was created for each threshold step and saved to the ROI manager. A threshold of 35–45 was set for the ipsilateral image, ensuring that the entire ipsilateral region was included and a mask was created using the selection tool and saved to the ROI. Using the ‘and’ option of the ROI tool, the mask of the ipsilateral region was overlaid with the contralateral mask and the level of overlap was measured in pixels for each threshold. The fraction of the ipsilateral region without overlap was then calculated for each contralateral threshold and reported as the fraction of segregated ipsilateral inputs. Segregation curves were plotted for each genotype and age.

### Barrel cortex anatomical analysis

P10 mice were euthanized and transcardially perfused with ice-cold PBS followed by 4% PFA in PBS. Brains were removed from the skull and postfixed in 4% PFA for 24 h. then, 50-µm brain sections were prepared on the tangential plane. Two different flattening techniques were used to analyze (1) the barrel area of the posteromedial barrel subfield and (2) the relative position of the posteromedial barrel subfield: (1) the two hemispheres were separated and the thalamus, HPC, entorhinal cortex and striatum were removed. Tissue was placed on a freezing block of optimal cutting temperature compound on the freezing microtome and gently flattened with a glass coverslip and 50-µm sections were prepared; (2) the two hemispheres were separated and the thalamus, HPC and part of the striatum were removed. Tissue was placed between the glass slides with capillary tubes as spacers and flattened. The tissue was fixed in 4% PFA for 24 h before processing. The flattened hemisphere was placed on the freezing microtome and cut (Bregma: 1.75 to −1.9 mm). To perform IHC, brain slices were placed in a well plate and washed with 0.1 M phosphate buffer and PBS. Slices were then blocked using 10% NGS, 0.3% Triton X-100 and 0.05% Na azide and PBS for 1 h at room temperature. Then, the primary antibody (1:1000 dilution VGLUT2, cat. no. 135-402-SY, 2B Scientific) solution containing 10% NGS, 0.3% Triton X-100 and 0.05% Na azide; PBS was added. Slices were incubated for 1 h at room temperature followed by 24 h at 4° C. The well plate was removed, the primary antibody solution was removed (plus 3× PBS washing) and allowed to come to temperature before the secondary antibody solution (10% NGS, 0.3% Triton X-100 and 0.05% Na azide and PBS) was added for 24 h. After this, slices were washed with PBS and 0.1 phosphate buffer for 1 h before being mounted.

### Analysis of dendritic spines and neuronal morphology

CA1 PCs were filled with biocytin during the electrophysiology experiments using a patch pipette. Slices were removed from the recording chamber and transferred to a 24-well plate containing 4% PFA for approximately 1 h after which they were transferred to PBS. Cells filled with biocytin were stained for streptavidin. The secondary antibody solution, consisting of 3% NGS, 0.1% Triton X-100, 0.05% Na azide and streptavidin Alexa Fluor 488 (1:500 dilution), was added and incubated for 24 h at 4 °C. Slices containing streptavidin-labeled CA1 PCs were imaged using the Zeiss 1800 confocal microscope. Images were acquired and analyzed blind to genotype. For the neuronal morphology analysis, individual PCs were imaged using a ×20 objective (NA = 0.8). Z-stacks of 2-µm step size were imaged along the length of the neuron and tiled together using ImageJ. For the spine density analysis, sections from three different dendritic compartments (apical oblique, apical tuft and basal) were imaged. Two to three dendrites were imaged for each compartment. Images were acquired with a ×63 oil immersion objective (NA = 1.4) using Nyquist sampling. For a section of dendrite, 2–3 Z-stacks with a 0.12 µm step were taken along the dendrite.

For neuronal morphology analysis, a 3D neuronal reconstruction of each cell was created by stitching images together using the tiling function of ImageJ. Images were imported into the Huygens Essentials software (Scientific Volume Imaging, http://svi.nl) and deconvolved using the fast CMLE algorithm, with SNR:20 and 100 iterations. Neuronal reconstructions of CA1 PCs were created. Tiled Z-stacks were imported into ImageJ; the Simple Neurite Tracer (SNT) plugin^52^ was used to create SWC trace files for each cell. Using the tracing tool, the length of dendrites was traced from the cell soma. By scrolling through the Z-stack, individual dendrites can be traced as they transverse through the slice. Trace files were converted into a 3D neuronal reconstruction representing individual cells. Neuronal reconstructions can be used to analyze dendritic complexity.

Neuronal morphology was assessed using Sholl analysis^53^. The number of dendritic intersections was quantified every 20-µm concentric distance from the cell soma. Using the SWC trace files, new trace files were created to account for the total number of dendrites and for each dendritic compartment. A Sholl analysis was performed using each of these trace files and the SNT. The total number of dendritic branching at a given distance from the soma was reported. To quantify the total length of dendrites in a given compartment, a path order analysis was carried out. The term ‘path order’ refers to the hierarchy of dendrites originating from the soma in respect to their branch order, that is, primary, secondary, tertiary. The ‘path order’ tool of SNT was used to produce a table including the numbers of primary, secondary and tertiary dendrites, and the length of each dendrite. Any dendrites above the tertiary hierarchy were recorded as tertiary dendritic paths. The total length and number of dendrites within each hierarchal compartment was reported.

For the spine density analysis, deconvolved images of dendritic sections were opened in ImageJ and the density of dendritic spines from apical oblique, apical tuft and secondary basal dendrites was quantified using the ‘Cell Counter’ plugin on ImageJ. The number of protrusions (spines) were manually counted from either side of the dendritic shaft starting from 5–10 µm from the branch point. The length of the dendritic section was measured and recorded using the ‘Measure’ tool.

### Calculating the astrocyte uptake of synaptic material

Images of the CA1 stratum radium were obtained using the Leica SP8 confocal microscope. Image stacks were acquired using Nyquist sampling with a z-step size of 0.12 µm and pixel size of 45 nm. Z-stack images of the CA1 stained with antibodies against SYP and GFAP were first preprocessed: digital images were first normalized to standardize intensity scales across the dataset. The SYP channels were clarified using a particle sharpen filter from OpenCV (Python package), while the GFAP channels were smoothed with a discrete Gaussian blur, reducing random noise and enhancing the meaningful signal. Image classification was conducted using a random forest algorithm in Vision4D, tailored through training based on 15 manually annotated sections each of SYP, GFAP and background signals. This model focuses on pixel-wise classification, relying on local contextual information without considering the overarching morphology of the labeled structures.

Segmentation outputs defining GFAP and SYP were encoded into TIFF format for in-depth analysis using Python v.3.9. To address z-stretching artifacts, operations from scipy.ndimage were used, including binary_erosion with generate_binary_structure to discern connected objects in the z-plane based on a parameterized structuring element. Minor GFAP elements below a volume threshold of 10 µm^3^ were excluded as noise using binary_closing. Synapses were filtered based on their cubic volume to between 0.01 µm^3^ and 1 µm^3^, based on previous quantitation of presynaptic bouton size in CA1 (ref. ^54^).

To quantify engulfment, 3D mesh models of GFAP and SYP structures were generated using marching_cubes from skimage.measure; surface areas were determined using mesh_surface_area. The regionprops function provided dimensional and positional descriptions of labeled regions, facilitating the analysis of synapse size and spatial relationships. Overlapping volumes between SYP and GFAP were quantified using distance_transform_edt from scipy.ndimage, identifying engulfment. Synaptic engulfment (100% engulfed) was then associated and analyzed in terms of relative GFAP volumes using spatial overlap metrics. Results were stored and manipulated with pandas DataFrame structures, which facilitated data handling and subsequent statistical testing.

### Induction of seizures

P17 and P20 mice were in injected intraperitoneally with the proconvulsant drug PTZ (cat. no. P6500, Merck) at a dose of 70 mg kg^−1^ in a volume of 10 ml kg^−1^. Mice were immediately placed into a 60 × 60 cm arena and recorded for a maximum of 10 min or when they reached a maximum severity level as assessed by the modified Racine scale^55^. Each severity level is characterized by a behavior and associated with a number from 0 to 7. A maximum time of 10 min was given; if the animals did not reach level 7 at this stage, they were removed and culled. No mice reached the maximum time. The experimenter was blind to genotype during the injection and analysis. For the analysis, videos were analyzed with the BORIS software. The latency to first seizure and inter-seizure interval time was recorded. The duration of each seizure was measured and the severity level was recorded.

### In vivo electrophysiology

Three-to-five-month-old *Csf1r*^∆FIRE/∆FIRE^ mice and their littermate controls were anesthetized with isoflurane (3% induction, 1% maintenance, 0.5 l min^−1^ in pure oxygen) and mounted into a stereotaxic frame. Two steel screws (1-mm diameter) were placed in small boreholes drilled above the cerebellum and olfactory bulb. A custom-made head-ring for later head-restraint training and recording was attached to the skull and the screws were tightly fixed using bone cement (Refobacin, Biomet). After surgery, mice were injected with carprofen (120–130 µl subcutaneously, 20 mg kg^−1^) and received a liquid supplement with 0.9% NaCl, 200–300 µl intraperitoneally. Mice were given at least 7 days to recover. Mice were then handled and habituated to head restraining on an air-cushioned styrofoam ball in a virtual reality (VR) system (JetBall, Phenosys). After habituation, mice were water-restricted (approximately 1.2 ml water per day) and trained to run in a VR linear treadmill for liquid rewards. When the animals could perform sufficient runs on the treadmill after 3–4 days, they were anesthetized and three small craniotomies were drilled, one above the PFC (anteroposterior: ≈1.9 mm; mediolateral: ≈0.5–1 mm) and two above the HPC in each hemisphere (anteroposterior: ≈−1.9 mm; mediolateral:≈ ±1.5 mm). The dura was removed. Craniotomies were sealed and protected using silicone (Kwik-Cast, World Precision Instruments). Animals were left to recover for at least 8 h before electrophysiological recordings started.

To perform the VR linear track treadmill task, mice were head-fixed on an air-cushioned styrofoam ball in a 270° TFT surround monitor system (JetBall, PhenoSys). Mice were trained to run to advance in a custom-made virtual linear corridor. The running path length was 50 cm for each trial. Once the animal reached the end of the virtual corridor in each trial, a 50-µl liquid reward (5% sucrose water) was delivered through a spout. After a 3-s inter-trial interval, animals were teleported to the start of the virtual corridor to initiate the subsequent trial. Each animal was trained once daily for 15–30 min for at least 3 days when sufficient amounts of liquid (≈1–1.2 ml) were obtained during the task. Trial signals in the tasks were recorded as transistor-transistor logic pulses by the acquisition device. The animal’s locomotion was detected by an XY-motion sensor at 50 Hz.

To perform recordings, mice were attached to the stereotaxic frame in the VR and were allowed to perform the linear treadmill task. Two silicone probes, each consisting of 128 channels on four parallel shanks (A4X32-Poly2–5mm-23s-200-177, NeuroNexus), were inserted into the PFC and the HPC, respectively through the craniotomies prepared. LFP signals were recorded using an Intan 128-channel head-stage from each probe and an Intan RHD recording controller. Signals were sampled at 20 kHz and digitized as 16-bit signed integers. All signal processing and data analysis were performed in MATLAB (MathWorks). The power-line interference (50, 100, 150 and 200 Hz) was removed from the LFP signals by a second-order band-stop filter. After eliminating further noise, LFP signals were downsampled to 2 kHz using a resample function that applies an FIR antialiasing low-pass filter. Then LFPs were filtered between 0.5 and 500 Hz using a second-order Butterworth band-pass filter. Segmentation of LFP data where the animal’s running speed was 5–10 cm s^−1^ were extracted for analysis. A 5-s window, backwards from 0.5 s before the reward delivery time, was selected in each trial. The power spectrum density and coherence analysis were obtained using the pwelch (window: 2 s; overlap: 50%) and the mscohere function (window: 2 s; overlap: 50%) functions, respectively. A theta (6–10 Hz):delta (2–4 Hz) ratio greater than 4 of the mean power spectral density in the HPC was applied to select clear running-associated and alertness-associated theta epochs as described previously^56–59^. Power was calculated as the sum of the power spectral density in each frequency band; coherence was calculated as the mean in each frequency band (theta: 4–12 Hz; beta: 15–25 Hz; gamma: 26–70 Hz).

### Statistics

Throughout the article, statistical tests are stated, along with *P* values and the test statistic used. Statistical tests (performed in Prism 10) were always two-sided and performed on biological replicates, not technical replicates. Multiple slices or cells measured from the same animal were treated as technical replicates. Data were generally assumed to be normal; when they failed the normality test, a nonparametric version of the *t*-test (Mann–Whitney *U*-test) was used. *T*-tests included Welch’s correction; otherwise, data variance was assumed to be similar. Differential expression analysis of RNA-seq data was performed using DESeq2, with a significance threshold calculated at a Benjamini–Hochberg-adjusted *P* < 0.05. Sample sizes ranged from 4 to 15 animals per condition or genotype. For cell counting and recording, the experimenter was blind to the condition or genotype. For all images displayed, contrast and brightness were applied in a linear manner across the entire image.

### Reporting summary

Further information on research design is available in the Nature Portfolio Reporting Summary linked to this article.

## Online content

Any methods, additional references, Nature Portfolio reporting summaries, source data, extended data, supplementary information, acknowledgements, peer review information; details of author contributions and competing interests; and statements of data and code availability are available at 10.1038/s41593-024-01833-x.

## Supplementary information

Reporting Summary

Supplementary Software 1 Supplementary Software 1 – MATLAB script function used to analyze the current step protocol.

Supplementary Software 2 Supplementary Software 2 – MATLAB script function used to calculate input resistance in response to −10 pA current injections.

Supplementary Data 1 Supplementary Data 1 – Differential gene expression analysis comparing expression in excitatory neurons in WT versus FIRE KO mice.

Supplementary Data 2 Supplementary Data 2 – Differential gene expression analysis comparing expression in inhibitory neurons in WT versus FIRE KO mice.

## Source data

Source Data Fig. 1 Statistical source data.

Source Data Fig. 2 Statistical source data.

Source Data Fig. 3 Statistical source data.

Source Data Fig. 4 Statistical source data.

Source Data Fig. 5 Statistical source data.

Source Data Fig. 6 Statistical source data.

Source Data Fig. 7 Statistical source data.

Source Data Fig. 8 Statistical source data.

## Data Availability

The accession codes related to the RNA-seq data (ArrayExpress) are E-MTAB-14156 and E-MTAB-14160. Data for the microglia-enriched genes are as described in ref. ^60^ and are publicly available (Sequence Read Archive accession no. SRP135960). Data that support the findings of this study are either available in the article and its supplementary materials, or from the authors upon reasonable request. Source data are provided with this paper.

## Code availability

Supplementary Software 1 and 2 custom MATLAB scripts are supplied as supplementary material.
