# Supplementary material for: Typical development of synaptic and neuronal properties can proceed without microglia in the cortex and thalamus
Source: Nat Neurosci. 2025 Jan 6;28(2):268–79. doi: 10.1038/s41593-024-01833-x (PMC11802452; doi:10.1038/s41593-024-01833-x)
Supplement: Supplementary file 1 — Reporting Summary [file 41593_2024_1833_MOESM1_ESM.pdf]

nature portfolio

Corresponding author(s): Giles Hardingham, Peter Kind

Last updated by author(s): 5/29/2024

Reporting Summary

Nature Portfolio wishes to improve the reproducibility of the work that we publish. This form provides structure for consistency and transparency in reporting. For further information on Nature Portfolio policies, see our Editorial Policies and the Editorial Policy Checklist.

Statistics

For all statistical analyses, confirm that the following items are present in the figure legend, table legend, main text, or Methods section.

n/a	Confirmed	
☐	☒	The exact sample size (*n*) for each experimental group/condition, given as a discrete number and unit of measurement
☐	☒	A statement on whether measurements were taken from distinct samples or whether the same sample was measured repeatedly
☐	☒	The statistical test(s) used AND whether they are one- or two-sided<br>*Only common tests should be described solely by name; describe more complex techniques in the Methods section.*
☐	☒	A description of all covariates tested
☐	☒	A description of any assumptions or corrections, such as tests of normality and adjustment for multiple comparisons
☐	☒	A full description of the statistical parameters including central tendency (e.g. means) or other basic estimates (e.g. regression coefficient) AND variation (e.g. standard deviation) or associated estimates of uncertainty (e.g. confidence intervals)
☐	☒	For null hypothesis testing, the test statistic (e.g. *F*, *t*, *r*) with confidence intervals, effect sizes, degrees of freedom and *P* value noted<br>*Give P values as exact values whenever suitable.*
☒	☐	For Bayesian analysis, information on the choice of priors and Markov chain Monte Carlo settings
☒	☐	For hierarchical and complex designs, identification of the appropriate level for tests and full reporting of outcomes
☒	☐	Estimates of effect sizes (e.g. Cohen's *d*, Pearson's *r*), indicating how they were calculated

Our web collection on statistics for biologists contains articles on many of the points above.

Software and code

Policy information about availability of computer code

Data collection	Illumina Novaseq 6000 platform; Leica LAS X 1.4.6;
Data analysis	ImageJ v1.53 was used for image analysis<br><br>RNA-seq reads were mapped to genome sequences using STAR v2.7.0f (Spliced Transcripts Alignment to a Reference, (Dobin et al., 2013)). Per-gene read counts were summarised using featureCounts v1.6.3 (Liao et al., 2014), and differential expression analysis performed using DESeq2 v1.24.0 (Love et al., 2014). Seurat R package, version 4.4.0 was used for single cell RNA-seq<br><br>Dobin, A., Davis, C.A., Schlesinger, F., Drenkow, J., Zaleski, C., Jha, S., Batut, P., Chaisson, M., and Gingeras, T.R. (2013). STAR: ultrafast universal RNA-seq aligner. Bioinformatics 29, 15-21.<br><br>Liao, Y., Smyth, G.K., and Shi, W. (2014). featureCounts: an efficient general purpose program for assigning sequence reads to genomic features. Bioinformatics 30, 923-930.<br><br>Love, M.I., Huber, W., and Anders, S. (2014). Moderated estimation of fold change and dispersion for RNA-seq data with DESeq2. Genome Biol 15, 550.<br><br>MATLAB was used for analysing electrophsiology data. Custom MATLAB scripts Supplementary Code 1 and Supplementary Code 2 are supplied as supplemental material.

For manuscripts utilizing custom algorithms or software that are central to the research but not yet described in published literature, software must be made available to editors and reviewers. We strongly encourage code deposition in a community repository (e.g. GitHub). See the Nature Portfolio guidelines for submitting code & software for further information.

Data

Policy information about availability of data

All manuscripts must include a data availability statement. This statement should provide the following information, where applicable:

- Accession codes, unique identifiers, or web links for publicly available datasets
- A description of any restrictions on data availability
- For clinical datasets or third party data, please ensure that the statement adheres to our policy

RNA-seq data has been deposited with ArrayExpress accession numbers E-MTAB-14156 and E-MTAB-14160. All data that support the findings of this study are either either available in the paper or available from the authors on request.

Research involving human participants, their data, or biological material

Policy information about studies with human participants or human data. See also policy information about sex, gender (identity/presentation), and sexual orientation and race, ethnicity and racism.

Reporting on sex and gender	n/a
Reporting on race, ethnicity, or other socially relevant groupings	n/a
Population characteristics	n/a
Recruitment	n/a
Ethics oversight	n/a

Note that full information on the approval of the study protocol must also be provided in the manuscript.

Field-specific reporting

Please select the one below that is the best fit for your research. If you are not sure, read the appropriate sections before making your selection.

☒ Life sciences ☐ Behavioural & social sciences ☐ Ecological, evolutionary & environmental sciences

For a reference copy of the document with all sections, see nature.com/documents/nr-reporting-summary-flat.pdf

Life sciences study design

All studies must disclose on these points even when the disclosure is negative.

Sample size

Sample size was estimated utilising 3R (Reduction, Replacement and Refinement) principles, with sample size numbers calculated to achieve satisfactory power based on the variance and effect size in published data [1-8].

1 Paolicelli, R. C. et al. Synaptic pruning by microglia is necessary for normal brain development. Science 333, 1456-1458, doi:10.1126/science.1202529 (2011).
2 Hoshiko, M., Arnoux, I., Avignone, E., Yamamoto, N. & Audinat, E. Deficiency of the microglial receptor CX3CR1 impairs postnatal functional development of thalamocortical synapses in the barrel cortex. J Neurosci 32, 15106-15111, doi:10.1523/JNEUROSCI.1167-12.2012 (2012).
3 Weinhard, L. et al. Microglia remodel synapses by presynaptic trogocytosis and spine head filopodia induction. Nat Commun 9, 1228, doi:10.1038/s41467-018-03566-5 (2018).
4 Stevens, B. et al. The classical complement cascade mediates CNS synapse elimination. Cell 131, 1164-1178, doi:10.1016/j.cell.2007.10.036 (2007).
5 Schafer, D. P. et al. Microglia sculpt postnatal neural circuits in an activity and complement-dependent manner. Neuron 74, 691-705, doi:10.1016/j.neuron.2012.03.026 (2012).
6 Favuzzi, E. et al. GABA-receptive microglia selectively sculpt developing inhibitory circuits. Cell 184, 5686, doi:10.1016/j.cell.2021.10.009 (2021).
7 Basilico, B. et al. Microglia shape presynaptic properties at developing glutamatergic synapses. Glia 67, 53-67, doi:10.1002/glia.23508 (2019).
8 Chu, Y. et al. Enhanced synaptic connectivity and epilepsy in C1q knockout mice. Proc Natl Acad Sci U S A 107, 7975-7980, doi:10.1073/pnas.0913449107 (2010).

Data exclusions

No data were excluded from the analysis.

Replication

All findings were replicated. Replication numbers are indicated within the figure legends. Replicates used for statistical purposes are biological replicates and not technical replicates, and all replications were included within the presented data.

Randomization	Since group allocation was based on genotype randomization was not needed or appropriate.
Blinding	Experimental analysis was performed blind to the genotype.

Reporting for specific materials, systems and methods

We require information from authors about some types of materials, experimental systems and methods used in many studies. Here, indicate whether each material, system or method listed is relevant to your study. If you are not sure if a list item applies to your research, read the appropriate section before selecting a response.

Materials & experimental systems

n/a	Involved in the study
☐	☒ Antibodies
☒	☐ Eukaryotic cell lines
☒	☐ Palaeontology and archaeology
☐	☒ Animals and other organisms
☒	☐ Clinical data
☒	☐ Dual use research of concern
☒	☐ Plants

Methods

n/a	Involved in the study
☒	☐ ChIP-seq
☐	☒ Flow cytometry
☒	☐ MRI-based neuroimaging

Antibodies

Antibodies used

Iba1, Abcam, ab283319 1:1000.
GFAP, Neuromics CH22102 1:1000.
Aldh1l1, Invitrogen, #702573 1:1000.
ACSA2 Miltenyi Biotec 130-116-245. 1:200
O4, Miltenyi Biotec 30-117-357, 1:200
CD11B BioLegend 101206, 1:500
CD45, BioLegend 103126, 1:100
Streptavidin Alexafluor 488, ThermoFisher, 1:500
Alexa Flour 488 goat anti-rabbit (1:500) (Invitrogen, A-11008)
Alexa Flour 647 goat anti-rabbit (1:500) (Invitrogen, A-21244)
Alexa Flour Plus 488 goat anti-rat (1:500) (Invitrogen, A-48262)

Validation

Iba1, Abcam, ab283319 1:1000. Picks up band of correct size in human monocytic leukemia monocyte lysate western but not control (MCF7). Labels microglia in human and rat brain slices. e.g. see Histol Histopathol. 2024 Jun;39(6):771-781. doi: 10.14670/HH-18-681

GFAP (Glial Fibrillary Acidic Protein) is strongly expressed in hippocampal astrocytes, and the GFAP antibody stains astrocytes in this region as well as reactive astrocytes elsewhere.
Burda, Joshua E et al. sorders.Nature vol. 606,7914 (2022): 557-564. doi:10.1038/s41586-022-04739-5
Ising, Christina et al. Nature vol. 575,7784 (2019): 669-673. doi:10.1038/s41586-019-1769-z

ALDH1L1:
This Antibody was verified by Relative expression to ensure that the antibody binds to the antigen stated. Antibody specificity was demonstrated by detection of differential basal expression of the target across the cell lines and tissues tested owing to their inherent genetic constitution.
Freitas-Andrade, Moises et al. Nature communications vol. 14,1 4965. 16 Aug. 2023, doi:10.1038/s41467-023-40682-3

ACSA2 Antibody raised against an astrocyte-specific surface epitope for FACS.
G. Kantzer, C. et al. (2017) Glia 65: 990–1004, Batiuk, M.Y. et al. (2017) J Biol. Chem. 292: 8874-8891

CD45 antibody for sorting of CD45-expressing immune cells. Validated by isolation of CD45+ cells from a mouse spleen cell suspension. Validated by comparing with other known clones recognizing the same antigen in a competition assay: REA737, I3/2.3, IBL-5/25

CD11B. Overlap in epitope recognition with cd11b antibody M1/70.15.11.5. Used for sorting CD11B-expressing immune cells.
Lebson, L. et al. (2010) J. Neurosci. 30 (29): 9651-9658, Liu, T. et al. (2014) Sci Rep 4: 4833

Animals and other research organisms

Policy information about studies involving animals; ARRIVE guidelines recommended for reporting animal research, and Sex and Gender in Research

Laboratory animals	Mice were used between the ages of 4 days and 5 months. The mice are on a mixed background of CBA and C56BL6/J. Mice were group-housed in environmentally-enriched cages within standard humidity and temperature controlled rooms (20C), with a 12-hour light dark cycle with free access to food and water. Ages of all mice used in experiments is stated in the legends.
Wild animals	No wild animals were used in this study.
Reporting on sex	The hypotheses we were testing on brain development were based on prior analysis of a mixed sex background so sex-specific analysis was not carried out.
Field-collected samples	No wild animals were used in this study.
Ethics oversight	All procedures described were performed either in the University of Edinburgh in compliance with the UK Animals (Scientific Procedures) Act 198b and University of Edinburgh Local Ethical Review Board

Note that full information on the approval of the study protocol must also be provided in the manuscript.

Flow Cytometry

Plots

Confirm that:

☒ The axis labels state the marker and fluorochrome used (e.g. CD4-FITC).

☒ The axis scales are clearly visible. Include numbers along axes only for bottom left plot of group (a 'group' is an analysis of identical markers).

☒ All plots are contour plots with outliers or pseudocolor plots.

☒ A numerical value for number of cells or percentage (with statistics) is provided.

Methodology

Sample preparation	Cerebral neocortices were collected then dissociated with Adult Brain Dissociation Kit (Miltenyi Biotec) according to the manufacturer's protocol on a gentleMACS Octo Dissociator using program 37C_ABDK_01. Dissociated samples were then treated with debris and red blood cell removal steps to obtain cell suspensions.
Instrument	FACSAria II Cell Sorter (BD Biosciences).
Software	flow cytometry software FCS Express
Cell population abundance	Cell abundance of microglia shown in Extended Data figure 1D
Gating strategy	Single cells were gated by side scatter (Height and Area) followed by side scatter (Height and Area). Dead cells were negatively selected using Draq7 and lymphocytes negatively selected by Ly6g. Microglia were defined as being high for CD11b and low/intermediate for Cd45.

☒ Tick this box to confirm that a figure exemplifying the gating strategy is provided in the Supplementary Information.
